# Lentinan-functionalized PBAE-G-nanodiamonds as an adjuvant to induce cGAS-STING pathway-mediated macrophage activation and immune enhancement

**DOI:** 10.1016/j.jpha.2023.12.012

**Published:** 2023-12-22

**Authors:** Zhiqiang Zhang, Li Wang, Xia Ma, Hui Wang

**Affiliations:** a College of Pharmacy, Henan University of Chinese Medicine, Zhengzhou, 450045, China; b Department of Traditional Chinese Medicine, Henan Agricultural University, Zhengzhou, 450046, China; c College of Animal Medicine, Henan University of Animal Husbandry and Economy, Zhengzhou, 450046, China

**Keywords:** LNT-PBAE-G-ND@OVA, Molecular mechanisms, cGAS-STING signaling

## Abstract

A series of biodegradable nanoparticle-based drug delivery systems have been designed utilizing poly(β-amino ester)-guanidine-phenylboronic acid (PBAE-G) polymers. In this study, a novel Lentinan-Functionalized PBAE-G-nanodiamond system was developed to carry ovalbumin (LNT-PBAE-G-ND@OVA). The impact of this drug delivery system on the activation and maturation of macrophages was then assessed. Furthermore, LNT-PBAE-G-ND@OVA induced potent antibody response and showed no obvious toxicity *in vitro* and *in vivo*. Moreover, treatment with LNT-PBAE-G-ND@OVA was sufficient to alter the expression of genes associated with the cGAS-STING pathway, and the LNT-PBAE-G-ND@OVA induced upregulation of costimulatory molecules. LNT-PBAE-G-ND@OVA treatment was sufficient to induce macrophage activation through a complex mechanism in which cyclic guanosine monophosphate-adenosine monophosphate (cGAMP) synthase (cGAS)-stimulator of interferon genes (STING) signaling plays an integral role.

## Highlights

• Innovative design and synthesis of LNT-PBAE-G-ND@OVA.
• LNT-PBAE-G-ND@OVA induced potent antibody response and showed no obvious toxicity *in vitro* and *in vivo*.
• LNT-PBAE-G-ND@OVA activation cGAS-STING signaling pathway plays an immune-enhancing role.

## Graphical abstract

Structural composition and the proposed mechanism of LNT-PBAE-G-ND@OVA.

**Figure:**
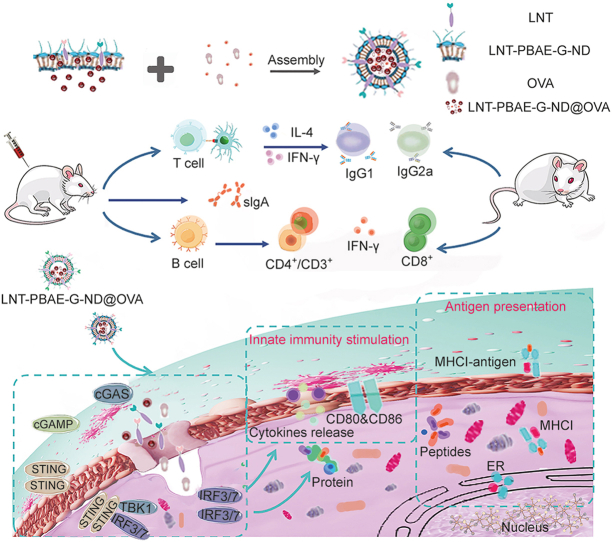

## 1 Introduction

Vaccination remains a reliable approach to efficiently preventing or mitigating a range of viral and bacterial diseases [1]. Relative to more traditional vaccines, protein subunit vaccines are sometimes viewed as being advantageous owing to the fact that they consist of highly purified components that can be clearly defined. They do not possess many of the native characteristics of actual pathogens, however, these protein subunit vaccines often induce less robust immune responses, necessitating the use of adjuvants to achieve effective immunogenicity and to regulate the resultant immune responses [2,3]. Aluminum-based adjuvants are the most frequently employed, allowing for the establishment of effective humoral immunity, although they are poorly equipped to enhance cellular immunity [4].

Lentinan (LNT) [5] is a key bioactive component of *Lentinula edodes*, and its potent immunomodulatory and antioxidant activities have led to increasing research interest in recent years [6,7]. Prior work suggests that LNT can stimulate the activation and maturation of a diverse array of immunological cell types such as lymphocytes, dendritic cells (DCs), natural killer (NK) cells, and macrophages [8]. By driving enhanced cytokine production, lymphocyte proliferation, macrophage and DC activation, and T cell responsivity, LNT can induce more effective immune responses. LNT is a β-1,3 β-glucan with a branching comb-like primary structure that harbors two β-1,6 glucosyl groups [9,10]. Critically, there is evidence supporting the ability of LNT to enhance immunity through functioning as a vaccine adjuvant [11]. LNT can stimulate the maturation, differentiation, and proliferation of key cells such as T lymphocytes, B cells, and macrophages, improve the balance of the host body, and achieve the restoration or enhancement of the reactivity of host cells to lymphocytes, hormones, and other bioactive factors. Owing to its inability to specifically target immune cells, however, LNT can be rapidly cleared from the body, highlighting the need for its targeted delivery using appropriate functional systems.

Nanodiamonds (NDs) are structures with extremely promising physicochemical characteristics that make them highly suitable for use in a range of settings, including excellent thermal and chemical stability, a range of functional groups, and a large surface area [12]. Accordingly, biomedical researchers have recently examined the utility of ND-based systems for vaccine delivery or drug carriers [13,14]. NDs are widely utilized for cellular imaging, as biosensory probes [15], or as photothermal agents owing to their satisfactory biocompatibility [16], unique mechanical properties [17], hydrophilic nature, and strong absorbance in the near-infrared (NIR) range [18]. NDs can serve as ideal drug delivery platforms owing to their combination of high surface area and many different functional groups [19]. Moreover, NDs can stimulate Toll-like receptor (TLR) signaling activity to more potently activate antigen-presenting cells (APCs) such as macrophages or DCs [20]. Given that physical adsorption or chemical modification can yield high ND loading ratios for antigens or other proteins of interest, these nanomaterials are also excellent candidates for vaccine delivery system design.

**Scheme 1:**
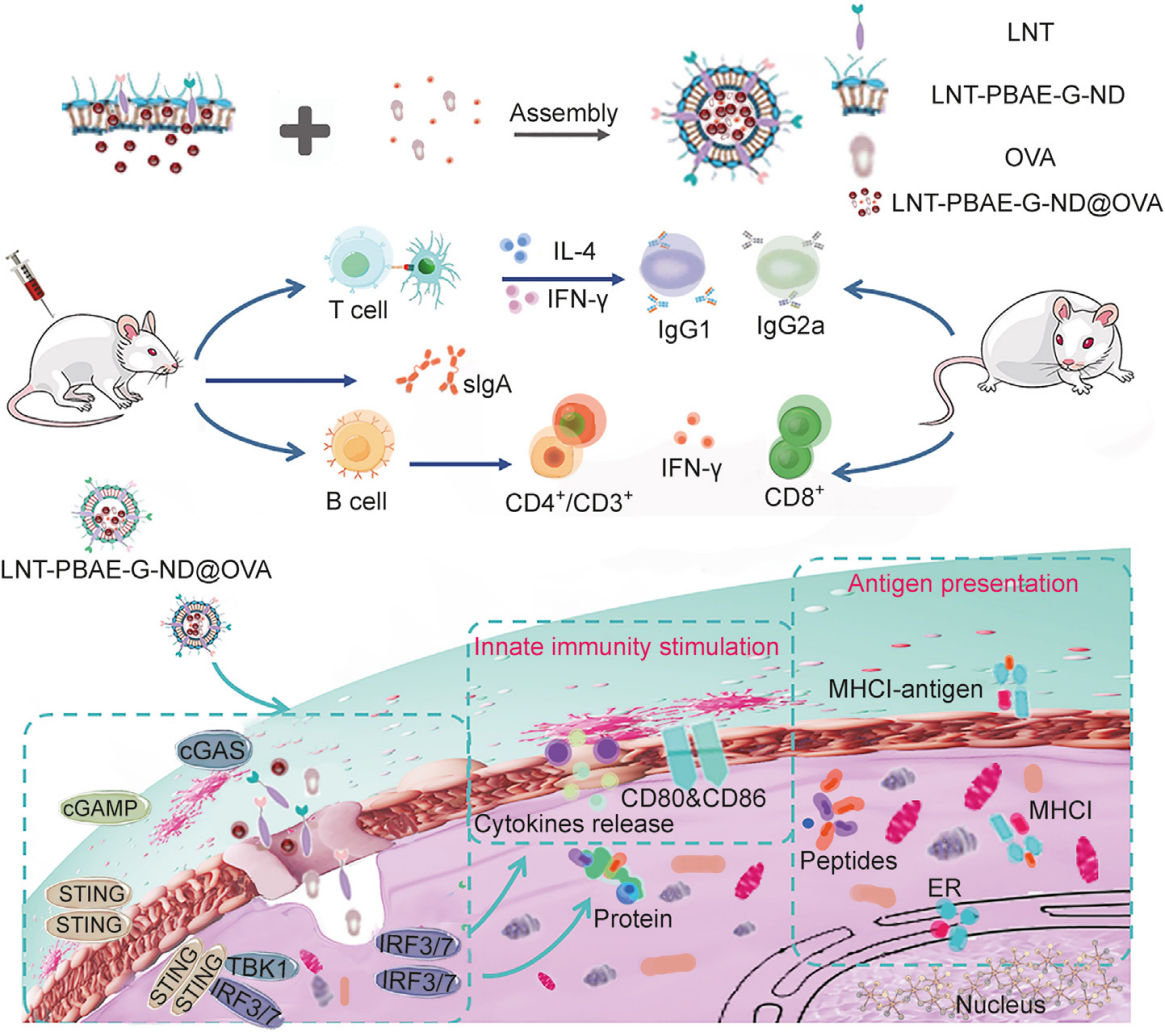
Schematic representation of the synthetic LNT-PBAE-G-ND@OVA. LNT-PBAE-G-ND@OVA: lentinan-functionalized PBAE-G-nanodiamond system was developed to carry ovalbumin; LNT: lentinan; PBAE-G: poly(β-amino ester)-guanidine-phenylboronic acid; ND: nanodiamond; OVA: ovalbumin; IL-4: interleukin 4; IFN-γ: interferon-γ; cGAS: cyclic guanosine monophosphate-adenosine monophosphate (cGAMP) synthase; STING: stimulator of interferon genes; TBK1: recombinant TANK binding kinase 1; IRF3/7: interferon regulatory factor 3/7; MHCI: major histocompatibility complex class I; ER; estrogen receptor.

The present study describes the use of a stimuli-responsive nanoparticle (NP) system with a core-shell structure as a platform for the encapsulation of a target antigen for use in immunization (Scheme 1). For this system, LNT was used to develop a biocompatible hydrophilic shell for these NPs that exhibited an excellent affinity for the lectins present on bacteria and macrophages, increasing NP bio-availability. The core of these NPs was comprised of biodegradable poly(β-amino ester)-guanidine-phenylboronic acid (PBAE-G) polymers amenable to the encapsulation of target antigens such as ovalbumin (OVA) under physiological conditions [21]. PBAE-G polymers can respond to high levels of reactive oxygen species (ROS) and low pH levels found in inflammatory settings, undergoing conversion into membrane permeable hydrophilic cationic polymers [22]. The PBAE-G polymer is converted to a hydrophilic polymer at a low pH due to the protonation of the tertiary amine in the polymer main chain. Moreover, the poly(β-amino ester)-guanidine-phenylboronic acid-bond (PBAE-G-B) polymer is converted to a hydrophilic PBAE-G polymer in the presence of ROS. The phenylboronic acid in the original PBAE-G polymer enhanced the hydrophobicity of the polymer and allowed the polymer to react with diols in polysaccharides via esterification [17]. The resulting phenylboronic ester linkage that connects the PBAE-G polymer and LNT is responsive to both low pH and ROS. This linkage is prone to dissociate in low pH, thereby promoting the disintegration of the NP. The oxidation of the B–C bond induced by ROS can lead to the decomposition of phenylboronic ester into 4-hydroxybenzyl alcohol and boric acid/ester, and convert the hydrophobic PBAE-G-B polymer into a hydrophilic form (PBAE-G) [18]. Taken together, the pH/ROS-triggered hydrophobic to hydrophilic transition of the PBAE-G-B polymer and disruption of the phenylboronic ester bonds destabilize the NP, causing the release of the cationic polymers and the OVA loaded inside of the NP.

The resultant NPs used in this study thus consisted of a core composed of ND-loaded OVA with an LNT adjuvant coating. Through the leveraging of the properties of these LNT-PBAE-G-ND materials, this system was able to enhance the activation of macrophages *in vitro* and to induce robust cellular and humoral immunity *in vivo*. The adjuvant used for these studies was highly stable and soluble with high levels of immunomodulatory and phagocytic activity. LNT-functionalized PBAE-G-nanodiamond system was developed to carry ovalbumin (LNT-PBAE-G-ND@OVA) treatment of mice led to the production of stronger cellular and humoral immunity, thus emphasizing the value of ND-based vaccine adjuvant systems.

## 2 Materials and methods

### 2.1 Reagents

Jiaozuo TianBao Material Technology Co., Ltd (Jiaozuo, China) was the source of the utilized NDs, while LNT was from Shanghai Yuanye Biotechnology Co., Ltd (Shanghai, China). Hydrazine hydrate was purchased from Sinopharm Chemical Reagent Co., Ltd (Ningbo, China). PBAE-G-B polymer was purchased from Aladdin Reagent Co., Ltd (Shanghai, China). TRIzol and fetal bovine serum (FBS) were purchased from Biyun Tian Biotech (Shanghai, Chian), while OVA and Cell Counting Kit-8 (CCK-8) kits and RU320521 (CAS No. 2262452-06-0) were purchased from Sigma-Aldrich (St. Louis, MO, USA). 4′,6-diamidino-2-phenylindole (DAPI) was purchased from Solarbio Science & Technology Co., Ltd., (Beijing, China). IgG, IgG2a, and IgG2b were purchased from ABclonal Biotechnology Co., Ltd (Wuhan, China). APC anti-mouse CD86 and FITC anti-mouse major histocompatibility complex class II (MHCII) were purchased from eBioscience, Inc. (San Diego, CA, USA). Rabbit monoclonal antibodies specific for cyclic guanosine monophosphate-adenosine monophosphate (cGAMP) synthase (cGAS) (E5V3W) (#79978), stimulator of interferon genes (STING) (D2P2F) (#13647), TANK-binding kinase-1 (TBK1)/NAK (E8I3G) (#38066), interferon regulatory factor-3 (IRF-3) (D6I4C) (#11904), and nuclear factor-kappa-B (NF-κB) p65 (D14E12) (#8242) were purchased from Cell Signaling Technology (Danvers, MA, USA).

### 2.2 LNT-PBAE-G-ND and LNT-PBAE-G-ND@OVA synthesis

A modified version of a protocol published previously was used for the synthesis of LNT-PBAE-G-ND [23]. LNT was then reacted with PBAE-G polymers for 8 h in dimethyl sulfoxide (DMSO), with a molecular sieve serving as a dehydrant, after which the mixture was filtered to produce a solution containing LNT-PBAE-G. Then, this solution was combined with NDs by preparing 50 mL of ethanol containing NDs (157 mg, 0.4 mmol) and 1-(3-Dimethylaminopropyl)-3-ethyl carbodiimide hydrochloride (EDC; 77 mg, 0.4 mmol), with this solution then being mixed for 30 min at 60 °C to facilitate ND carboxyl group activation followed by the dropwise addition of this mixture to the LNT-PBAE-G (149 mg) solution that had been warmed to 60 °C. The mixture was combined for 24 h, after which it was added to a dialysis bag with a 4.5 kDa molecular weight cut-off (MWCO), followed by exchange with pure H_2_O for 48 h to eliminate any unreacted EDC or LNT-PBAE-G-ND from the mixture. Samples were then lyophilized, with ethanol being used to wash the crude LNT-PBAE-G-ND product three times to facilitate unreacted ND removal.

Drug-loaded NPs were prepared by suspending 300 mg of polymer components in 3 mL of DMSO and then adding 10 mg of OVA to this solution. The resultant mixture was then gradually combined with 15 mL of phosphate buffered saline (PBS; pH 8.0) and mixed vigorously, followed by dialysis (MWCO: 4.5 kDa) against PBS (pH 7.4) to remove all organic solvents. The solvent was replaced once at a 12 h interval, and for three times in total. This same approach was also used for empty NP preparation.

### 2.3 Characterization and stability of LNT-PBAE-G-ND@OVA

Scanning electron microscopy (SEM) was used to assess the morphology of LNT-PBAE-G-ND@OVA preparations (Model S-4800 II FESEM, Hitachi High-Technologies (China) Co., Ltd, Shenzhen, China). Dynamic light scattering (DLS) was used to assess NP size and Zeta potential values. LNT binding efficiency in LNT-PBAE-G-ND preparations was assessed using a phenol-sulfuric acid approach, with binding efficiency (EE%) being determined as follows:

EE% = (1−Q1/Q2)×100%

where Q2 corresponds to the amounts of LNT and OVA, while Q1 corresponds to the amount of unbound LNT and OVA.

LNT-PBAE-G-ND@OVA sample stability was assessed by dissolving the freeze-dried LNT-PBAE-G-ND@OVA in ddH_2_O and then assessing Zeta potential and particle size as above.

### 2.4 OVA and LNT release assay

The release of OVA and LNT at inflammatory microenvironment was studied. Macrophages were harvested from Institute of Cancer Research (ICR) mice as reported previously [23]. Macrophages cells incubated with LNT-PBAE-G-ND@OVA and interleukin (IL)-6 at concentration (10 μg/mL) for 4, 8, 12, 24, 48, 96, 192 and 384 h to observe the OVA and LNT release ratio. The released content of OVA was quantified using the bicinchoninic acid (BCA) assay kit, while the released content of LNT was measured using the phenol-sulfuric acid method.

### 2.5 Cytotoxicity analyses

Macrophages were harvested from ICR mice as reported previously [23]. These cells were then treated with a range of LNT-PBAE-G-ND@OVA or LNT-PBAE-G-ND concentrations, with Dulbecco's modified Eagle medium (DMEM) serving as a control. At appropriate time points, cells were rinsed using PBS, and then a CCK-8 kit was used to assess cytotoxicity as reported previously, with absorbance being analyzed at 450 nm using a microplate reader. Viability was determined relative to the control group (%).

### 2.6 Macrophage surface marker analyses

Flow cytometry was used to assess CD80, CD86, and MHCII (Cell Signaling Technology, Danvers, MA, USA) expression on macrophage surfaces. Cells were treated with DMEM (negative control), lipopolysaccharide (HY-D1056, MCE, Shanghai, China) (LPS; 500 ng/mL, positive control), LNT-PBAE-G-ND@OVA (10 μg/mL), OVA (10 μg/mL), or LNT (10 μg/mL). After 48 h, cells were stained with appropriate antibodies and analyzed with a flow cytometer (C6, Becton, Dickinson and Company, New York, NY, USA).

### 2.7 Macrophage uptake assays

The efficiency of LNT-PBAE-G-ND@OVA uptake was determined by adding macrophages to 6-well plates and treating them with LNT-PBAE-G-ND@OVA (10 μg/mL) or LNT-PBAE-G-ND (10 μg/mL). After washed with PBS for three times, cells were fixed for 15 min in 4% paraformaldehyde, and nuclei were stained for 5 min with DAPI (50 μL). LNT-PBAE-G-ND@OVA uptake efficiency was then determined via confocal laser scanning microscopy.

### 2.8 Murine immunization

For this study, BALB/c mice (females, 6 weeks old, *n* = 12) were obtained from the Comparative Medicine Centre of Zhengzhou University. Mice were housed per the guide for the care and use of laboratory animal issued by the Henan University of Animal Husbandry and Economy IACUC,with this protocol having received IACUC approval (Approval No: 2021BAD34B02). Mice were subcutaneously immunized with saline, OVA, LNT, LNT/OVA, LNT-PBAE-G-ND, or LNT-PBAE-G-ND@OVA, with both LNT and OVA being used at the same concentration (500 μg/mL), while LNT-PBAE-G-ND and LNT-PBAE-G-ND@OVA doses were determined based on binding rates (674.6 μg/mL, 697.2 μg/mL). After 14 days, mice received an intramuscular booster injection with 0.2 mL of an equivalent dose of the appropriate treatment. Blood samples were obtained on days 14, 28, and 42 following this second immunization, while on day 42, mice were euthanized and major organs were harvested. Blood was drawn from the orbital sinus, and 600 −800 μL of blood was taken from each mouse.

### 2.9 Enzyme-linked immunosorbent assay (ELISA) test

ELISA test was used to analyze murine serum samples to detect concentrations of OVA-specific IgG, IgG2a and IgG2b on days 14, 28, and 42 following the second immunization. ELISA test was also used to measure inflammatory cytokine levels. Absorbance at 450 nm was assessed via microplate reader. Samples were assessed in quadruplicate.

### 2.10 Lymphocyte immunophenotyping

On day 28 following the second round of immunization, T cell subpopulations were analyzed by collecting splenic lymphocytes from mice. These cells were then added to 24-well plates (1 × 10^6^/well) for 48 h, after which they were harvested and stained in the dark for 30 min with anti-CD3e-FITC, anti-CD4-APC, and anti-CD8a-PE at 4 °C. Cells were then rinsed three times using PBS, fixed using 4% paraformaldehyde, and analyzed via flow cytometry. Analyses were conducted in triplicate.

### 2.11 RNA-seq assay

Macrophages were added to 6-well plates (1 × 10^6^/well) and treated with LNT-PBAE-G-ND@OVA (10 μg/mL) in triplicate, as above, with unsupplemented DMEM serving as a control. TRIzol (Ambion, Beijing, China) was used to extract RNA from these cells after 48 h. The Illumina Hiseq4000 platform (Baimaike Technology Inc., Beijing, China) was then used for RNA-seq analyses (PE 150).

### 2.12 Quantitative polymerase chain reaction (qPCR)-based validation

RNA-seq results were verified by randomly selecting 9 different genes, the expression of which was then confirmed via qPCR using HiScript® II Q RT SuperMix for qPCR (+gDNA wiper) and ChamQ™ SYBR® qPCR Master Mix (Vazyme Biotech Co., Ltd., Nanjing, China) based on provided directions. Table 1 lists the primers for these 9 different genes and β-actin. Data were analyzed via the 2^−ΔΔCt^ approach.

**Table 1:** Size, Zeta-potential, LNT-grafting efficiency and OVA-grafting efficiency of ND, LNT-PBAE-G-ND, and LNT-PBAE-G-ND@OVA.

Nanoparticles	Size (nm)	Zeta-potential (mV)	LNT-grafting efficiency (%)	OVA-grafting efficiency (%)
ND	27.78 ± 0.87	8.13 ± 1.21	–	–
LNT-PBAE-G-ND	121.50 ± 12	−14.10 ± 0.98	65.43	–
LNT-PBAE-G-ND@OVA	145.10 ± 8.47	−17.78 ± 1.05	63.21	76.21

LNT-PBAE-G-ND@OVA: lentinan-functionalized PBAE-G-nanodiamond system was developed to carry ovalbumin; LNT: lentinan; PBAE-G: poly(β-amino ester)-guanidine-phenylboronic acid; ND: nanodiamond; OVA: ovalbumin.

### 2.13 Western immunoblotting

Macrophages were added to 6-well plates (1 × 10^6^/well) and treated with LNT-PBAE-G-ND@OVA (10 μg/mL) in triplicate, as above, with unsupplemented DMEM serving as a control. Radio immunoprecipitation assay (RIPA) buffer (Biyun Tian Biotech) was used to extract protein from samples following a 48 h treatment, followed by dilution with loading buffer to equivalent protein concentrations and heating for 10 min at 95 °C. Proteins were separated via sodium dodecyl sulfate-polyacrylamide gel electrophoresis (SDS-PAGE), transferred to polyvinylidene fluoride (PVDF) membranes, and these blots were blocked for 2 h using 5% non-fat milk followed by overnight incubation at 4 °C with appropriate primary antibodies. Following being washed three times using PBST), blots were then incubated for 2 h with goat anti-rabbit IgG horseradish peroxidase (HRP), and washed three more times. An enhanced chemiluminescence (ECL) kit (Biosharp life sciences, Beijing, China) was used for protein detection.

### 2.14 cGAS inhibition assay

Macrophages were added to 6-well plates (1 × 10^6^/well), followed by pretreatment for 4 h with RU320521 (37.5 μM) to inhibit cGAS. Cells were then treated for an additional 48 h with LNT-PBAE-G-ND@OVA (10 μg/mL). Cells not treated with AG490 inhibitor were subject to treatment for 48 h with LNT-PBAE-G-ND@OVA (10 μg/mL). Supernatant IL-12, IL-1β, and tumor necrosis factor-α (TNF-α) levels as well as the expression of CD40, CD80, and CD86 on cells positive for CD11c expression were assessed as above.

### 2.15 Statistical analysis

Data were means ± standard error of measurement (SEM), and were compared via Duncan's multiple range test. *P* < 0.05 was the significance threshold.

## 3 Results and discussion

### 3.1 LNT-PBAE-G-ND production and characterization

**Fig. 1:**
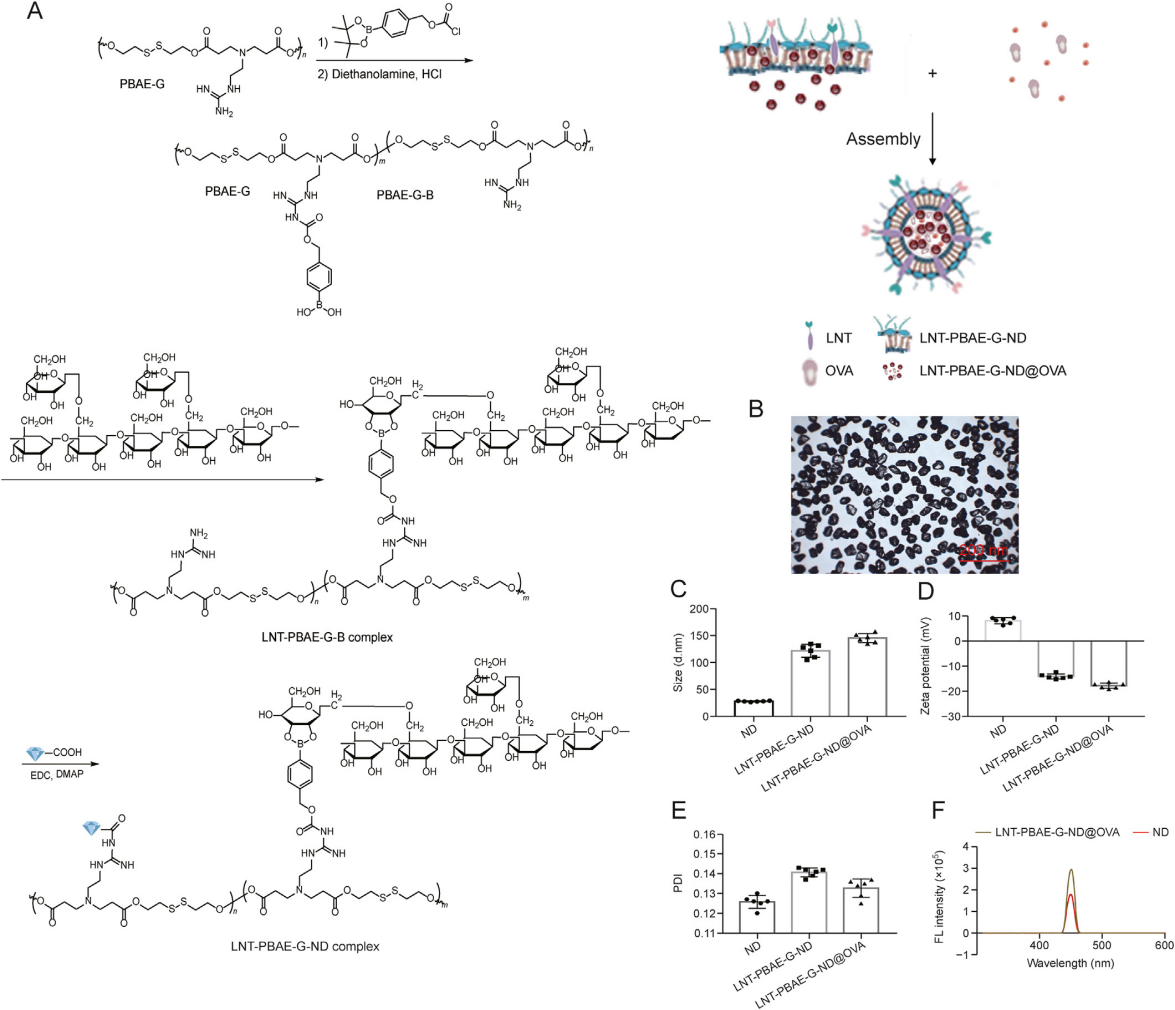
Lentinan (LNT)-poly(β-amino ester)-guanidine-phenylboronic acid (PBAE-G)-nanodiamond (ND) production and characterization. (A) The strategy used to prepare PBAE-G-ND and LNT-PBAE-G-ND. (B, C) LNT-PBAE-G-ND preparations were characterized via transmission electron microscopy (B) and dynamic light scattering (C). (D) Zeta-potential and (E) polymer dispersity index (PDI) values were assessed for LNT-PBAE-G-ND preparations. (F) Normalized ND and fluorescent (FL) spectra in H_2_O. OVA: ovalbumin; EDC: 1-(3-Dimethylaminopropyl)-3-ethyl carbodiimide hydrochloride; DMAP: 4-Dimethylaminopyridine.

PBAE-G-B polymers were prepared as reported previously (Fig. 1A) [23]. A blasting method was used to generate pristine ND NPs, with sonication increasingly yielding small nano-scale NDs, thus obtaining the final NPs with a size of approximately 25 nm LNT-PBAE-G-ND@OVA mixed with 30 mg of prepared polymer and 3.0 mg of OVA in 300 μL PBS. The core-shell structure of these LNT-PBAE-G-ND@OVA NPs was confirmed via transmission electron microscopy (TEM) (Figs. 1B and C, and Table 1), with final NPs being ∼145.1 nm in size (Fig. S1). Negative Zeta-potential values were observed for both LNT-PBAE-G-ND (−14.1 ± 0.98 mV) and LNT-PBAE-G-ND@OVA (−17.78 ± 1.05 mV) (Fig. 1D), and all of these NPs presented with low polymer dispersity index (PDI) values were consistent with low levels of polydispersion (Fig. 1E). ^1^H nuclear magnetic resonance (NMR) spectrum of LNT, PBAE-G-P, LNT-PBAE-G in D_2_O are shown in Fig. S2. Fluoresent (FL), Fourier transform infrared spectroscopy (FTIR), and UV-vis spectra of ND were measured in solutions containing both LNT-PBAE-G-ND and LNT-PBAE-G-ND@OVA (Figs. 1F, S3 and S4). A phenol-sulfuric acid method was used to assess solutions used to synthesize LNT-PBAE-G-ND@OVA containing unbound LNT, enabling the calculation of LNT binding efficiency. Overall, 65.43% and 63.21% of LNT was found to be successfully bound to the respective LNT-PBAE-G-ND and LNT-PBAE-G-ND@OVA preparations (Table 1), with this excellent polysaccharide loading likely being attributable to the fact that NDs exhibit a high surface area.

### 3.2 NP stability

**Fig. 2:**
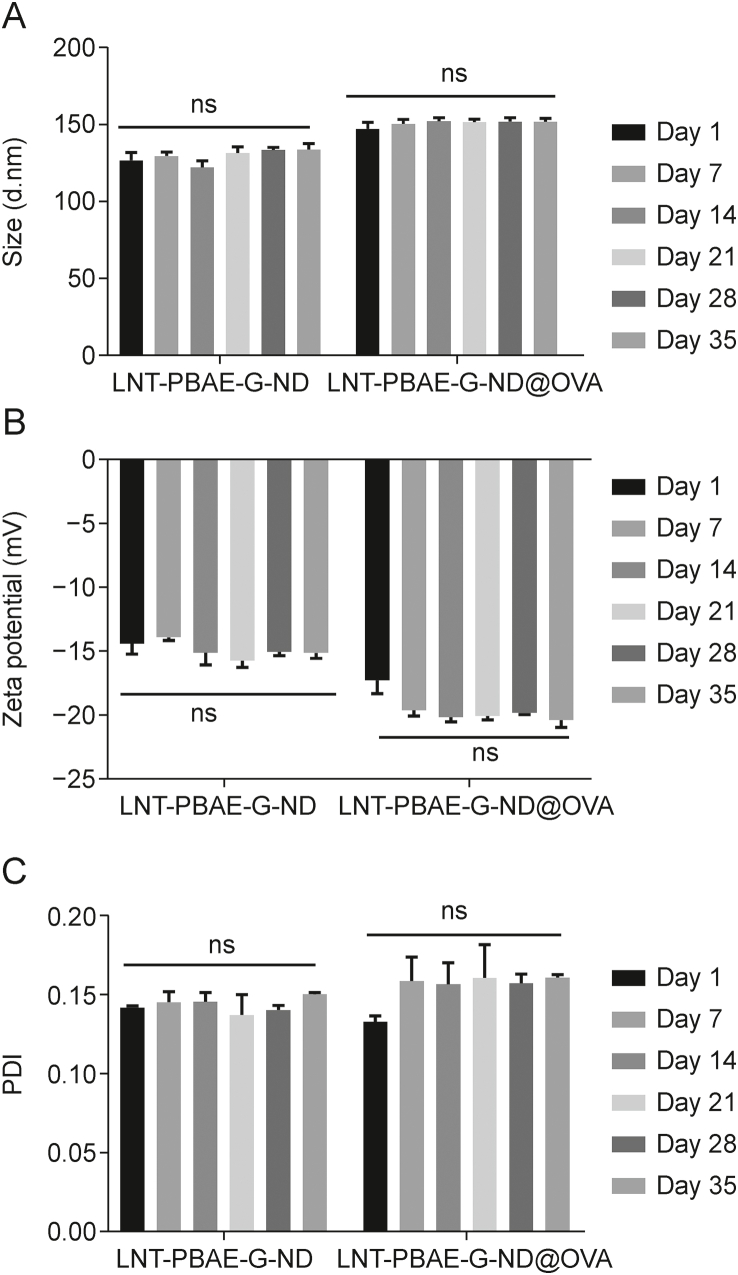
Lentinan (LNT)-poly(β-amino ester)-guanidine-phenylboronic acid (PBAE-G)-nanodiamond (ND) and LNT-PBAE-G-ND@ovalbumin (OVA) stability. Following freeze-drying and dissolution, (A) hydrodynamic size, (B) Zeta potential), and (C) polymer dispersity index (PDI) of LNT-PBAE-G-ND and LNT-PBAE-G-ND@OVA samples in H_2_O were assessed, revealing no differences among any of these groups. ns: not significant.

LNT-PBAE-G-ND and LNT-PBAE-G-ND@OVA NP sample stability was examined by measuring the PDI, particle size, and Zeta potential values for these particles over a 4-week period of incubation at 4 °C (Table 1, and Fig. 2). The LNT-PBAE-G-ND@OVA particles remained roughly 145–170 nm in size from day 1 to 35 (*P* > 0.05), whereas LNT-PBAE-G-ND sheets remained 120–140 nm in size, with smaller sized corresponding to successful LNT modification resulting in greater hydrated particle size (Fig. 2A). Zeta-potential and PDI values remained negatively charged and unchanged from day 1 to 35 for both LNT-PBAE-G-ND and LNT-PBAE-G-ND@OVA preparations (Figs. 2B and C, *P* > 0.05). As such, both LNT-PBAE-G-ND and LNT-PBAE-G-ND@OVA are highly stable after repeated rounds of dissolution and freeze-drying.

### 3.3 The release of LNT and OVA from prepared NPs

**Fig. 3:**
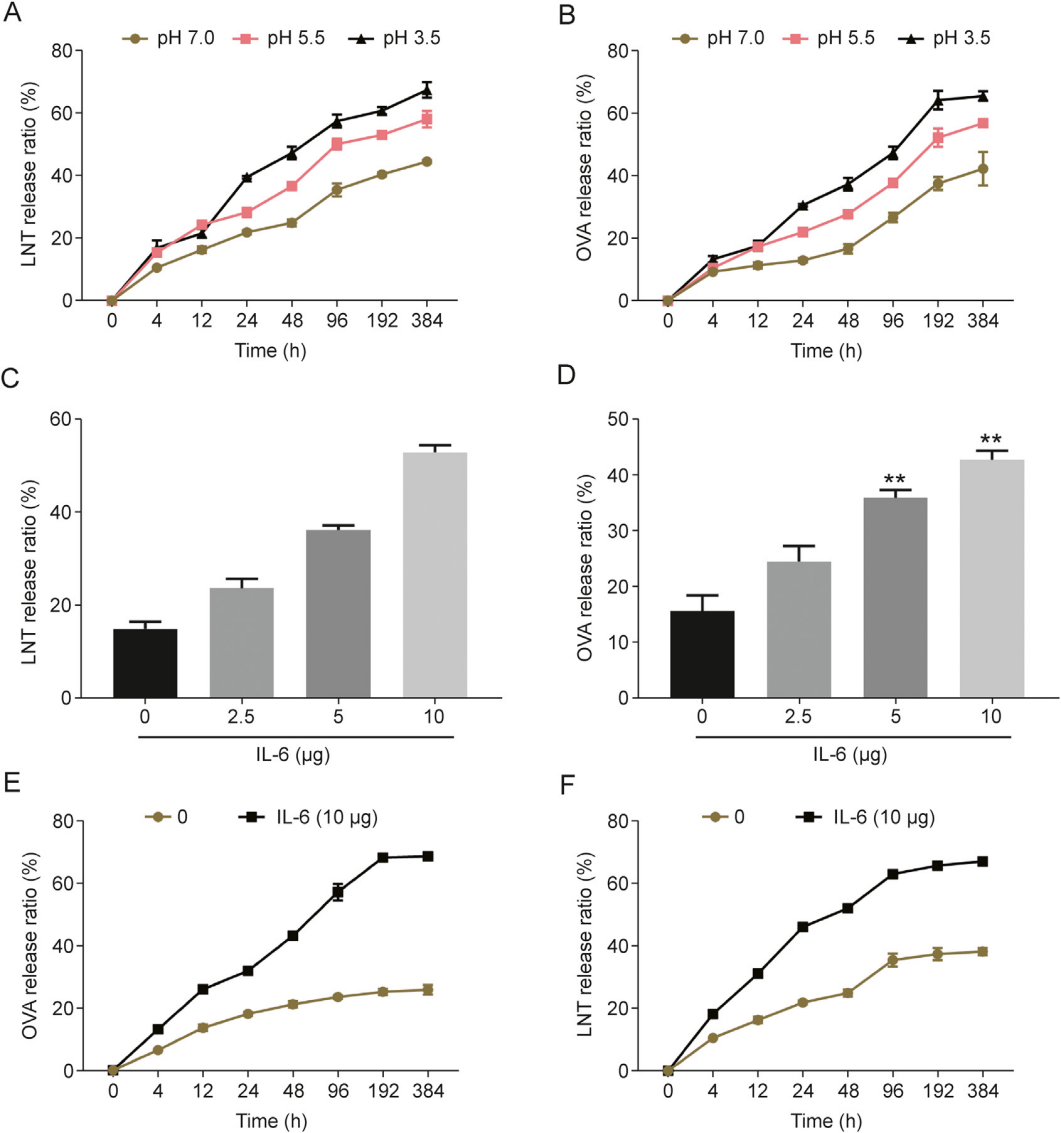
Analysis of the release of lentinan (LNT) and ovalbumin (OVA) from prepared nanoparticles (NPs). (A, B) The release profiles of LNT (A) and OVA (B) were assessed at the indicated pH levels. The dialysis systems were incubated in a 37 °C shaker and 100 μL samples outside the dialysis bag were collected at different time intervals (4, 12, 24, 48, 96, 192, 384 h) for LNT and OVA analysis. (C–F) Murine neutrophils were activated with interleukin (IL)-6, after which LNT (C and F) and OVA (D and E) release profiles from prepared NPs were assessed. 100 μL samples were collected at different time intervals (4, 12, 24, 48, 96, 192, 384 h) for LNT and OVA analysis.

When prepared, PBAE-G-B polymers can respond to high ROS levels and low pH values, both of which are commonly observed under inflammatory microenvironmental conditions and drive the conversion of these polymers into hydrophilic cationic forms thereof [24]. Vaccine adjuvants regulate inflammatory signaling pathways in an immunostimulatory fashion such that LNT-PBAE-G-ND may be highly responsive to inflammatory conditions in a manner conducive to immunity [25]. At pH of 3.5, LNT and OVA were more rapidly released from LNT-PBAE-G-ND@OVA preparations than they were at pH of 7.0 (Figs. 3A and B). When a neutrophil inflammation model was established, high levels of LNT and OVA were rapidly released from LNT-PBAE-G-ND@OVA in the inflammatory microenvironment (Figs. 3C−F). The morphology of the NP was observed under transmission electron microscope. It was found that NPs degraded with the increase of the concentration of hydrogen peroxide (Fig. S5).

### 3.4 Analyses of the cytotoxicity and uptake of LNT-PBAE-G-ND@OVA when used to treat macrophages

**Fig. 4:**
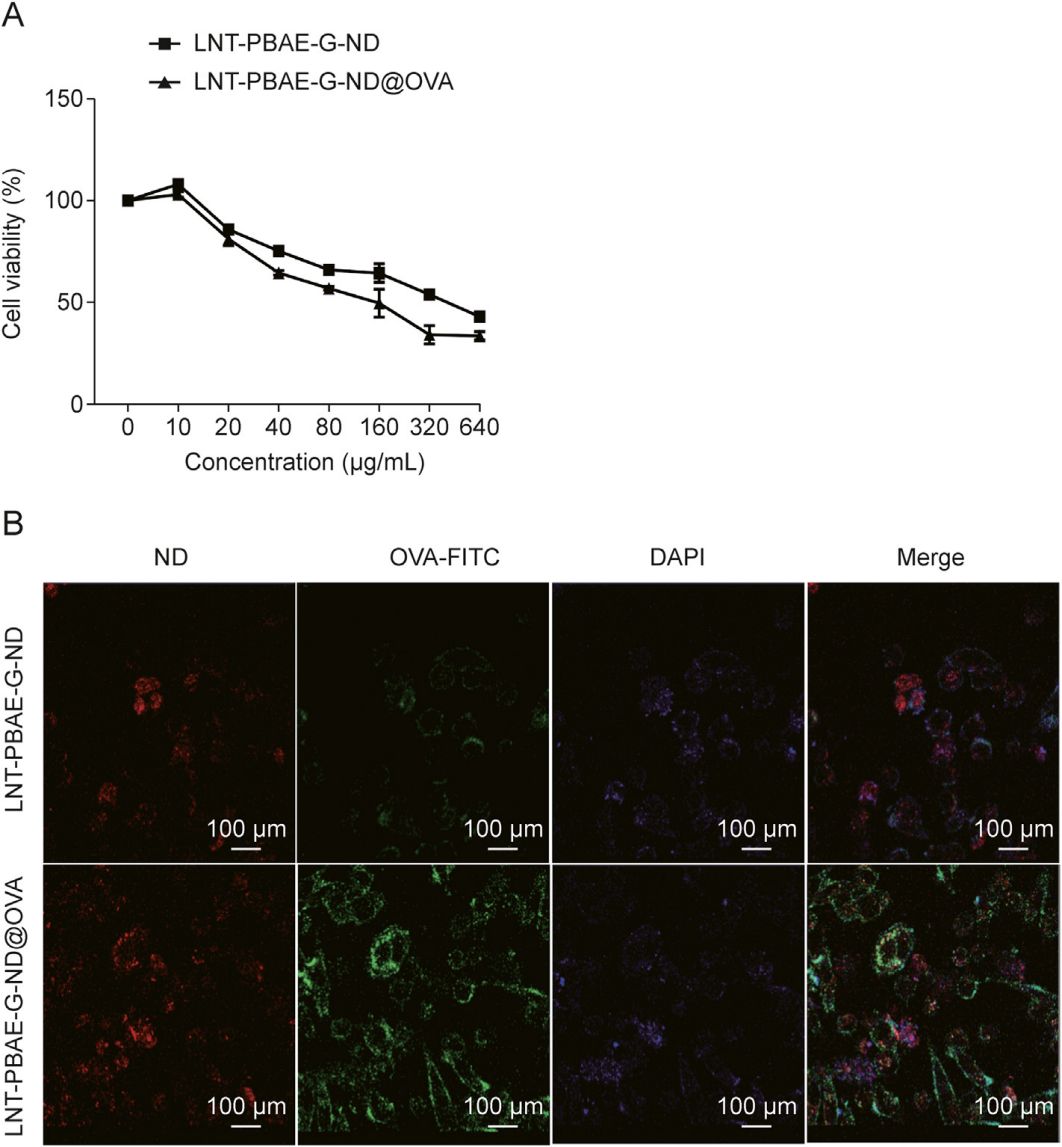
The uptake and cytotoxicity of lentinan (LNT)-poly(β-amino ester)-guanidine-phenylboronic acid (PBAE-G)-nanodiamond (ND)@ovalbumin (OVA) when used to treat macrophages. (A) Macrophage viability was examined following a 48 h treatment using LNT-PBAE-G-ND and LNT-PBAE-G-ND@OVA. (B) Following treatment with LNT-PBAE-G-ND or LNT-PBAE-G-ND@OVA, macrophages were examined via confocal laser scanning microscopy (OVA-FITC: green; DAPI: blue; ND: red). FITC: fluorescein isothiocyanate; DAPI: 4′,6-diamidino-2-phenylindole.

A CCK-8 assay was used to examine the cytotoxic effects of LNT-PBAE-G-ND@OVA or LNT-PBAE-G-ND treatment of macrophages following exposure for 48 h to a range of concentrations of these preparations. At the 40 μg/mL dose level, the viability of macrophages is over 74.6%. At a concentration of 10 μg/mL, both LNT-PBAE-G-ND and LNT-PBAE-G-ND@OVA were able to promote macrophage proliferation (Fig. 4A). As such, all future experiments were performed at the 10 μg/mL dose level.

The ability of macrophages to readily take up antigens is required for their ability to stimulate subsequent immunity [26], and both macrophages and neutrophils serve as early responders in the innate immune system that act to rapidly destroy invading pathogens [27]. The internalization of LNT-PBAE-G-ND and LNT-PBAE-G-ND@OVA by macrophages was examined via confocal microscopy, revealing that OVA-FITC and NDs were readily internalized (Fig. 4B), confirming the efficient ability of LNT-PBAE-G-ND@OVA to be taken up by these APCs.

### 3.5 Macrophage activation marker expression

**Fig. 5:**
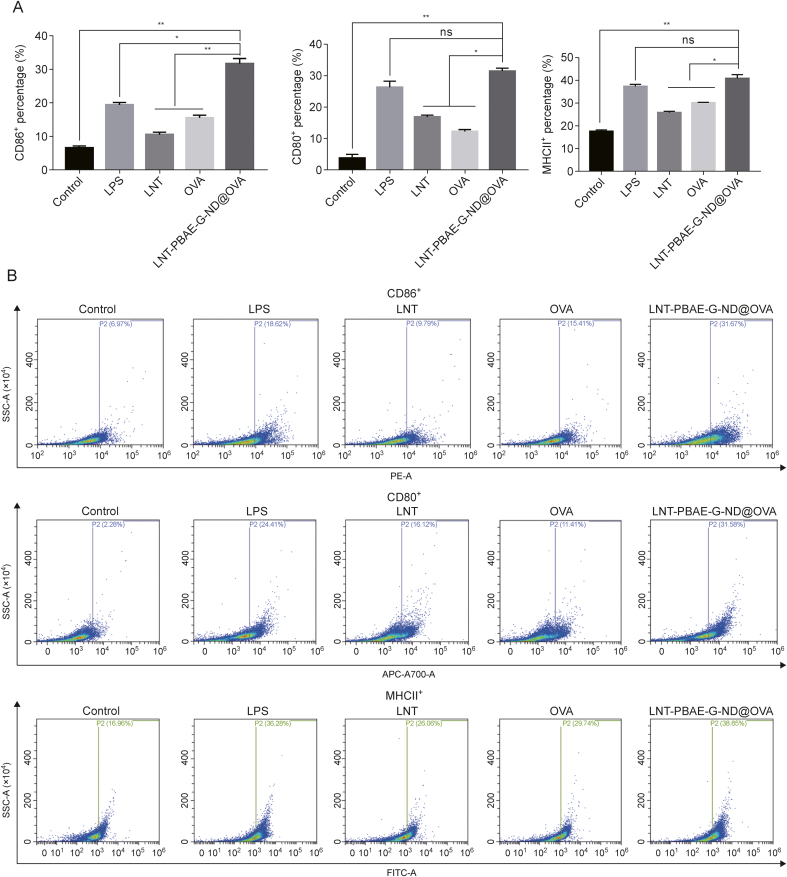
The impact of lentinan (LNT)-poly(β-amino ester)-guanidine-phenylboronic acid (PBAE-G)-nanodiamond (ND)@ovalbumin (OVA) treatment on the activation of macrophages. (A) Macrophage CD80, CD 86 and major histocompatibility complex class II (MHCII) expression. (B) Representative flow plots.

Following the internalization of pathogen-derived proteins, macrophages become activated such that they can present these antigens to T cells, activating adaptive immunity [28]. Macrophages are thus essential for both cellular and humoral immune response orchestration [29], with high levels of costimulatory markers such as CD80, CD86, and MHCII being expressed following activation, allowing these cells to promote the activation of T cells [30]. Accordingly, flow cytometry was used to assess CD80, CD86, and MHCII expression by macrophages treated with LNT-PBAE-G-ND@OVA. The marked upregulation of all three of these costimulatory markers was observed following LNT-PBAE-G-ND@OVA treatment relative to the control group (*P* < 0.05), reaching levels comparable to those in the LPS positive control group (*P* > 0.05, Fig. 5).

### 3.6 Macrophage cytokine secretion

**Fig. 6:**
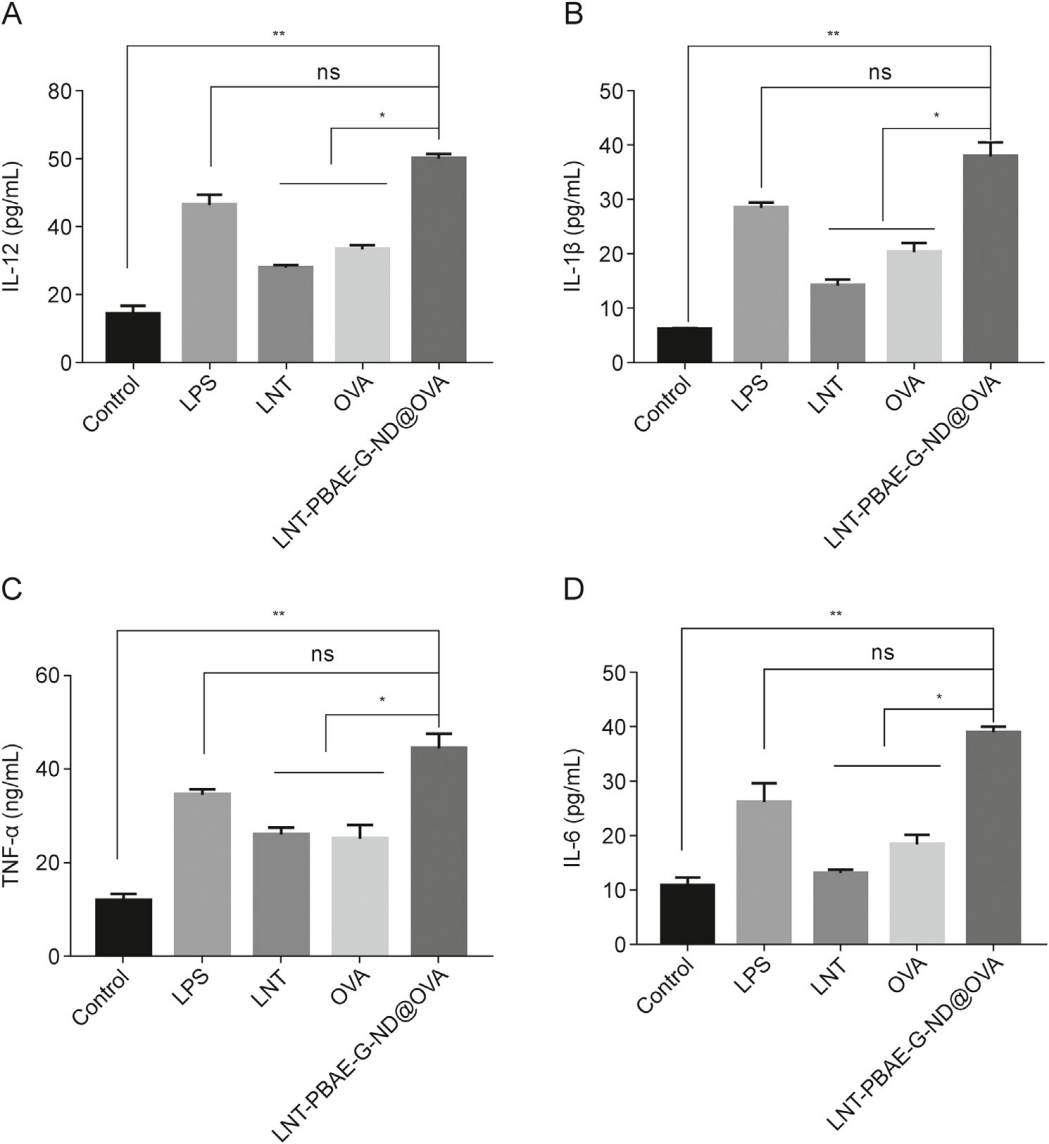
Macrophage cytokine secretion. (A) Interleukin (IL)-12, (B) IL-1β, (C) tumor necrosis factor-α (TNF-α) and (D) IL-6concentrations released by macrophages. Data are means ± standard error of measurement (SEM), *n* = 3, ^∗^*P* < 0.05, ^∗∗^*P* < 0.01, ns: not significant. LPS: lipopolysaccharide; LNT: lentinan; OVA: ovalbumin; LNT-PBAE-G-ND@OVA: lentinan-functionalized poly(β-amino ester)-guanidine-phenylboronic acid-nanodiamond system was developed to carry ovalbumin.

IL-12 is a pro-inflammatory cytokine that is important for T cell proliferation and interferon-γ (IFN-γ) production [31]. When the phagocytose antigens and undergo activation, macrophages can release a range of inflammatory cytokines including IL-1β and IL-12 [32]. Accordingly, macrophage activation was further assessed by analyzing supernatant IL-1β, IL-6, IL-12, and TNF-α levels upon LNT-PBAE-G-ND@OVA treatment. Consistent with the above data, higher levels of all four of these cytokines were released following stimulation with LNT-PBAE-G-ND@OVA (*P* < 0.05 or *P* < 0.01, Fig. 6). These data suggest that LNT-PBAE-G-ND@OVA NPs can readily activate macrophages.

### 3.7 Analyses of serum antibody production in response to LNT-PBAE-G-ND@OVA

**Fig. 7:**
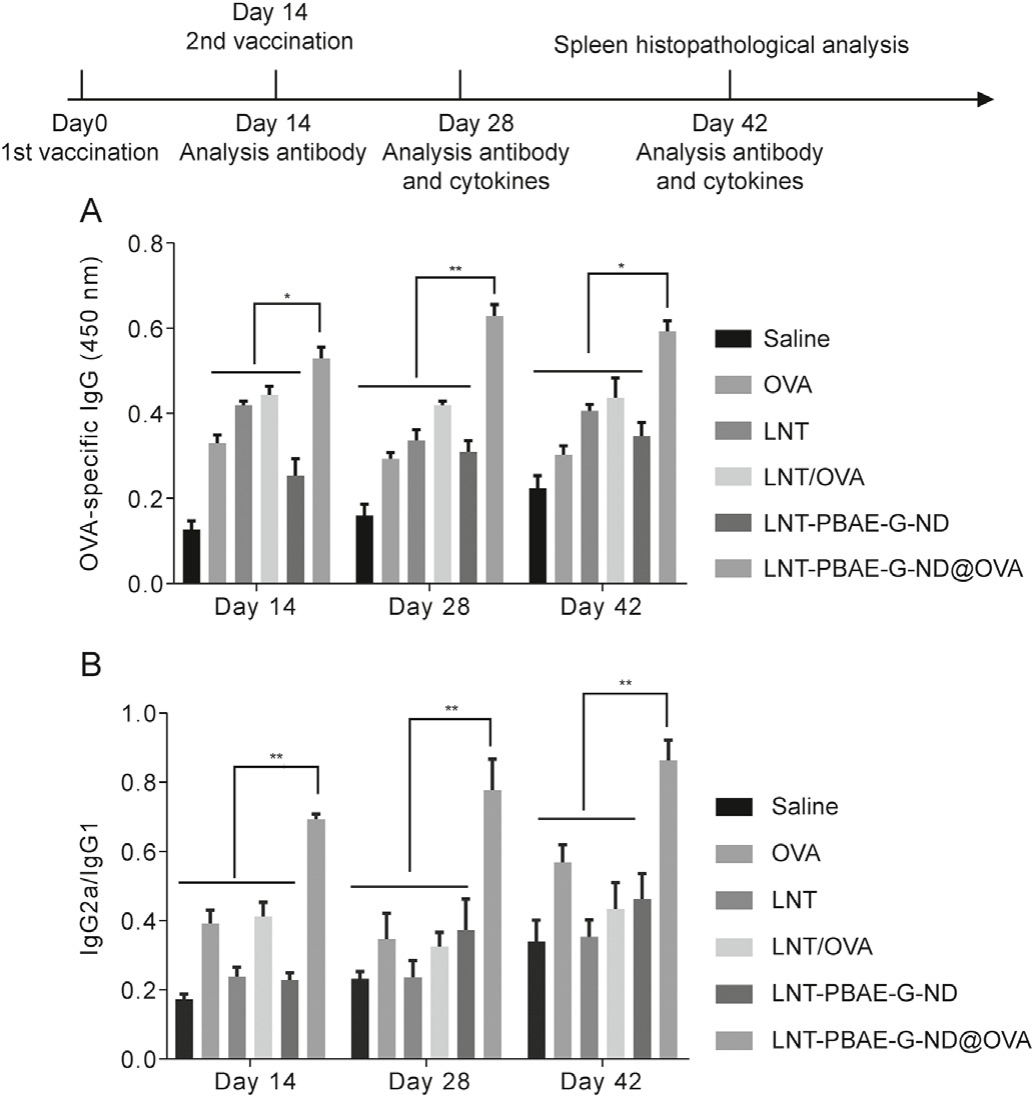
Schematic overview of the employed experimental approach. Mice received a primary immunization on day 0, followed by booster immunization on day 14. On days 14, 28, and 42, mice were sacrificed and serum samples were collected to measure antibody levels, with cytokine levels also being assessed on days 28 and 42. (A) Ovalbumin (OVA)-specific IgG levels were assessed on the indicated days. (B) IgG1 and IgG2a levels were measured on the indicated days. Data are means ± standard error of measurement (SEM), *n* = 3, ∗*P* < 0.05, ^∗∗^*P* < 0.01. LNT: lentinan; OVA: ovalbumin; LNT-PBAE-G-ND: lentinan-functionalized poly(β-amino ester)-guanidine-phenylboronic acid-nanodiamond; LNT-PBAE-G-ND@OVA: lentinan-functionalized poly(β-amino ester)-guanidine-phenylboronic acid-nanodiamond system was developed to carry ovalbumin.

Antibody responses can be used to gauge the impact of LNT-PBAE-G-ND@OVA treatment on the adaptive immune response [33], and the concentrations of IgG, IgG2a, and IgG2b were therefore measured via ELISA. Significantly increased IgG levels were observed following LNT-PBAE-G-ND@OVA treatment relative to treatment with LNT, OVA, LNT-PBAE-G-ND, or LNT/OVA alone on days 28 and 42 post-immunization (Fig. 7). Treatment with LNT-PBAE-G-ND@OVA also resulted in the release of higher IgG1 levels relative to those observed in other treatment groups, while IgG2a responses to LNT-PBAE-G-ND@OVA administration were more robust than those driven by OVA treatment (*P* < 0.01). Adjuvants can alter the relative induction of Th1 and Th2 CD4^+^ T cell responses, with the measurement of different serum antibody titers offering insight into these responses [34,35]. Specifically, IgG1 is the primary antibody subtype associated with Th2 immunity, whereas IgG2a and IgG2b are the main antibody types associated with Th1 cellular immune responses [36]. These results are thus consistent with the ability of LNT-PBAE-G-ND@OVA treatment to induce more potent humoral and cellular immunity.

### 3.8 Effects on T lymphocyte subpopulations

**Fig. 8:**
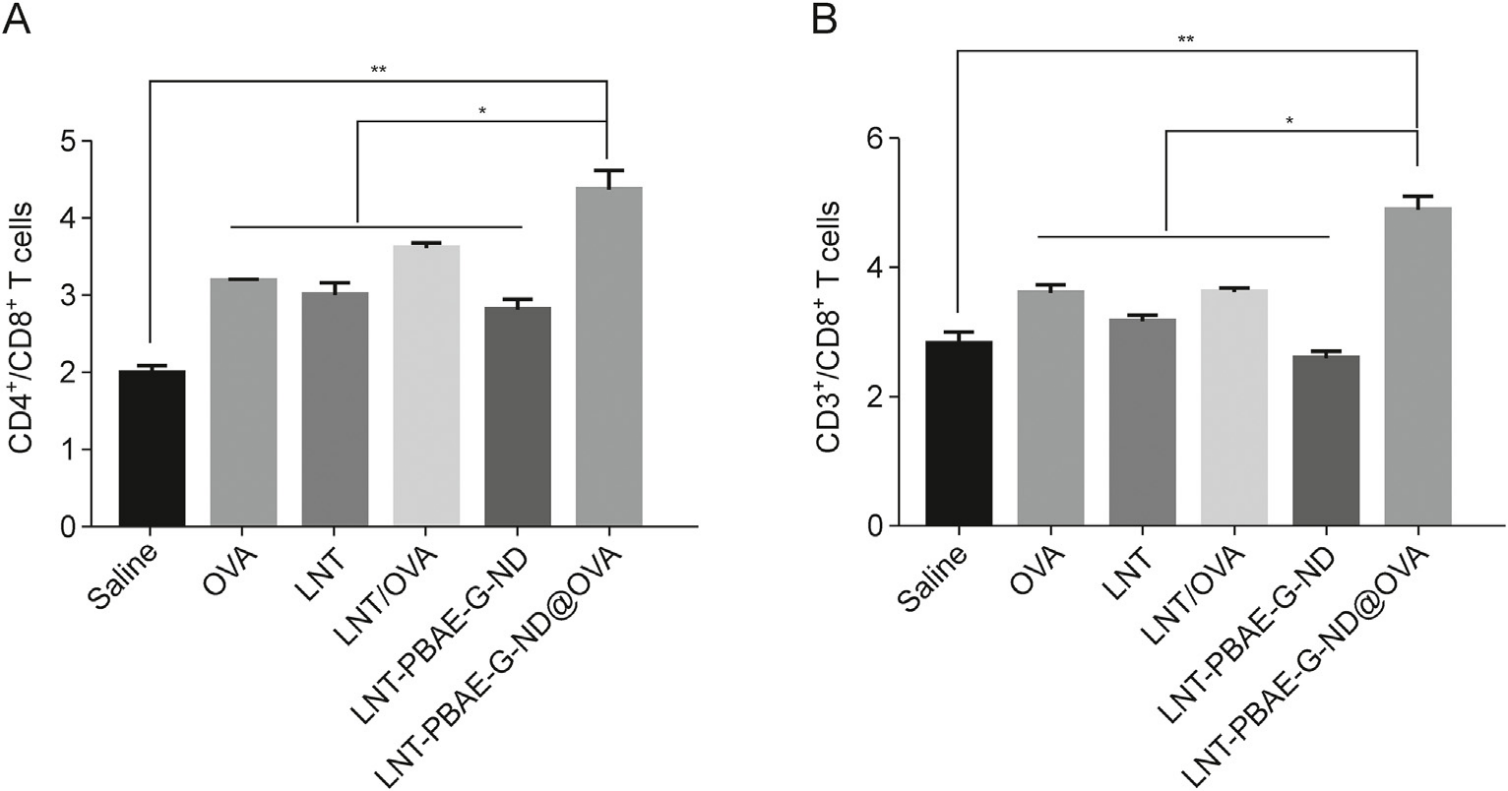
Changes in T cell subpopulation ratios. Flow cytometry was used to assess relative (A) CD4^+^ and (B) CD3^+^ to CD8^+^ T cell ratios in splenocytes collected from mice on day 42 after immunization with the indicated preparations. Data are means ± standard error of measurement (SEM), *n* = 3, ∗*P* < 0.05, ^∗∗^*P* < 0.01LNT: lentinan; OVA: ovalbumin; LNT-PBAE-G-ND: lentinan-functionalized poly(β-amino ester)-guanidine-phenylboronic acid-nanodiamond; LNT-PBAE-G-ND@OVA: lentinan-functionalized poly(β-amino ester)-guanidine-phenylboronic acid-nanodiamond system was developed to carry ovalbumin.

On day 42 following immunization, splenocytes were harvested from these mice and analyzed via flow cytometry to assess the ratios of different T cell subpopulations (CD4^+^/CD8^+^ and CD3^+^/CD8^+^ T cells). Overall, LNT-PBAE-G-ND@OVA treatment was associated with higher CD4^+^ and CD3^+^ to CD8^+^ T cell ratios relative to other tested treatments (Fig. 8), consistent with the ability of this platform to induce robust *in vivo* cellular immunity.

### 3.9 Serum cytokine levels

**Fig. 9:**
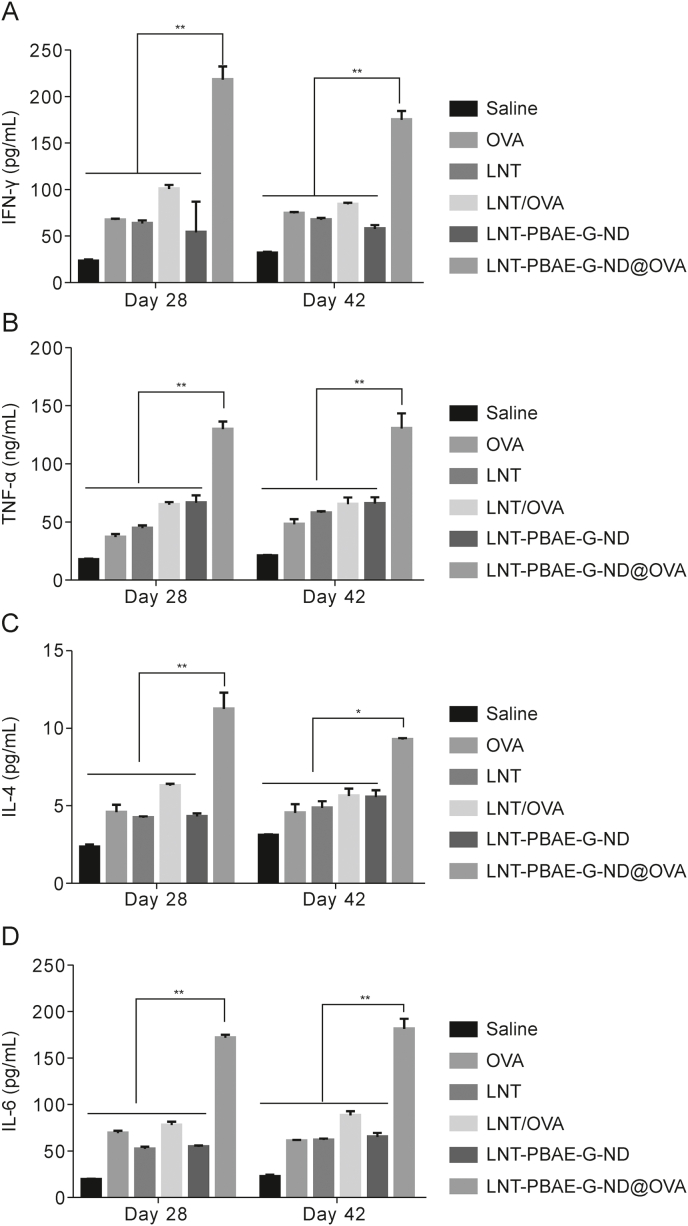
Serum concentrations of Th1 cytokines including (A) interferon-γ (IFN-γ) and (B) tumor necrosis factor-alpha (TNF-α) (B), as well as Th2 cytokines including (C) interleukin (IL)-4 and (D) IL-6 in immunized mice were examined via enzyme-linked immunosorbent assay (ELISA). Data are means ± standard error of measurement (SEM), *n* = 3, ∗*P* < 0.05, ^∗∗^*P* < 0.01. LNT: lentinan; OVA: ovalbumin; LNT-PBAE-G-ND: lentinan-functionalized poly(β-amino ester)-guanidine-phenylboronic acid-nanodiamond; LNT-PBAE-G-ND@OVA: lentinan-functionalized poly(β-amino ester)-guanidine-phenylboronic acid-nanodiamond system was developed to carry ovalbumin.

ELISA was further used to measure serum cytokine levels on days 28 and 42 after primary immunization [37]. Treatment with LNT-PBAE-G-ND@OVA led to the production of higher levels of Th1 cytokines (IFN-γ and TNF-α) relative to other treatments (*P* < 0.05) (Fig. 9). TNF-α is important for the induction of cellular Th1-based immunity [38]. Similarly, IL-4 and IL-6 were produced at higher levels following LNT-PBAE-G-ND@OVA treatment relative to other treatments (*P* < 0.05). These data suggest that LNT-PBAE-G-ND@OVA immunization can induce robust Th1 and Th2 immune responses, in line with the above antibody data.

### 3.10 Analyses of the *in vivo* biosafety of LNT-PBAE-G-ND@OVA

**Fig. 10:**
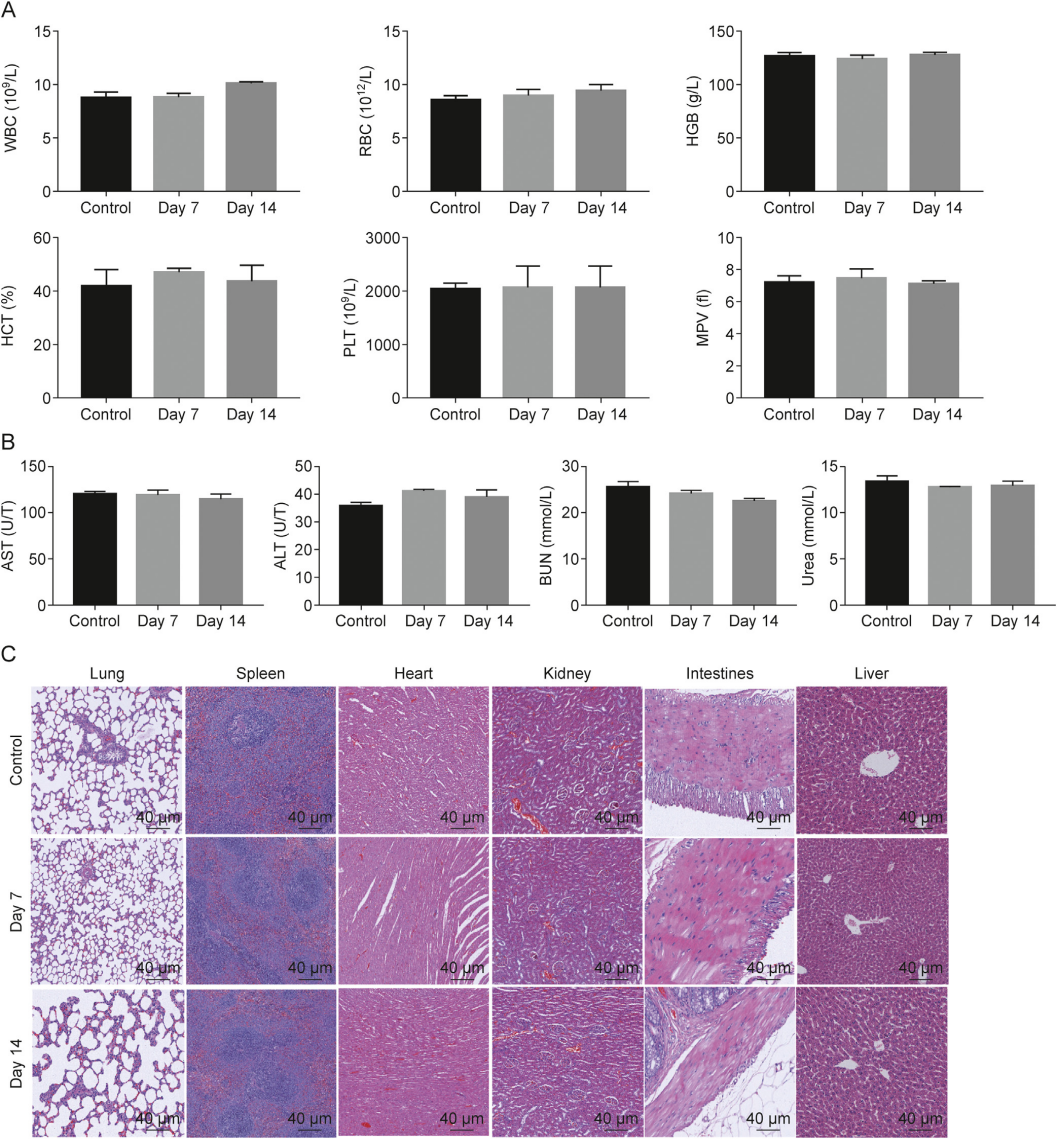
Analyses of the *in vivo* biosafety of lentinan-functionalized poly(β-amino ester)-guanidine-phenylboronic acid-nanodiamond system was developed to carry ovalbumin (LNT-PBAE-G-ND@OVA). (A) Hematological indices in mice on days 7 and 14 post-immunization.(B) Serum biochemical indicators (alanine amino transferase (ALT), aspartate amino transferase (AST), blood urea nitrogen (BUN) and urea) were analyzed in mice on days 7 and 14 post-immunization. Data are means ± standard error of measurement (SEM), *n* = 3. (C) Hematoxylin and eosin (H&E) staining was used to analyze samples of lung, spleen, heart, kindy, intestines, and livertissue from mice on days 7 and 14 post-immunization. WBC: white blood cells; RBC: red blood cells; HGB: hemoglobin; HCT: hematocrit; PLT: platelets; MPV: mean platelet volume.

An effective vaccine adjuvant must exhibit an excellent biosafety profile. Accordingly, the ability of LNT-PBAE-G-ND@OVA to induce major organ toxicity was next examined. Blood biochemical indices were unchanged following treatment with this adjuvant candidate (Figs. 10A and B), and there was no difference in serum biochemical markers of renal or liver function detected after treatment. Similarly, hematoxylin and eosin (H&E) staining of major organs (lungs, liver, spleen, kidneys, heart) did not reveal any significant histological changes following LNT-PBAE-G-ND@OVA treatment, with no evidence of inflammation or related tissue damage (Fig. 10C).

### 3.11 LNT-PBAE-G-ND@OVA promotes immunostimulatory cGAS-STING-TBK1-IRF3 pathway activation

**Fig. 11:**
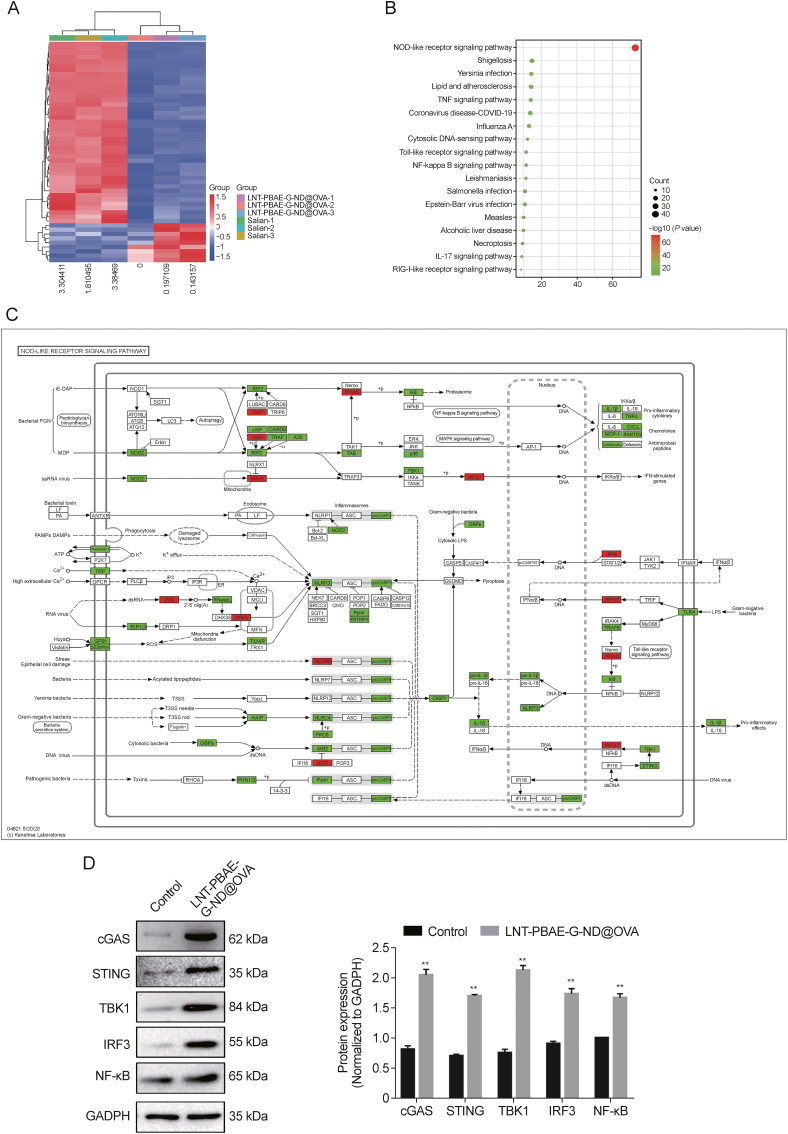
Changes in macrophage mRNA expression following treatment for 48 h with lentinan-functionalized poly(β-amino ester)-guanidine-phenylboronic acid-nanodiamond system was developed to carry ovalbumin (LNT-PBAE-G-ND@OVA). (A) DEGs were identified when comparing control and lentinan-functionalized poly(β-amino ester)-guanidine-phenylboronic acid-nanodiamond (LNT-PBAE-G-ND)-treated cells were compared via hierarchical clustering heat maps, with red and green respectively being used to identify genes that were upregulated and downregulated. (B) Differentially expressed genes(DEGs) were subject to Kyoto Encyclopedia of Genes and Genomes (KEGG) pathway enrichment analyses. (C) Significantly enriched genes in the NOD-like pathway, with upregulation and downregulation being respectively shown in green and red, respectively. (D) LNT-PBAE-G-ND@OVA promoted cyclic guanosine monophosphate-adenosine monophosphate (cGAMP) synthase-stimulator of interferon genes-recombinant TANK binding kinase 1-interferon regulatory factor 3 (cGAS-STING-TBK1-IRF3) pathway activation in macrophages, as measured via Western immunoblotting for cGAS, STING, TBK1, and IRF3, with relative protein expression being shown. Data are means ± standard error of measurement (SEM), *n* = 3, ^∗∗^*P* < 0.01 GADPH: glyceraldehyde-3-phosphate dehydrogenase.

To better understand the ability of the interplay between LNT-PBAE-G-ND@OVA and macrophage activation, an RNA-Seq analysis of these macrophages was performed. The top 30 most upregulated and downregulated differentially expressed genes (DEGs) are compiled in Table S1, and heat maps of hierarchically clustered DEGs when comparing cells treated with saline or LNT-PBAE-G-ND@OVA are shown in Fig. 11A. Kyoto Encyclopedia of Genes and Genomes (KEGG) pathway analyses of these DEGs revealed several enriched pathways following LNT-PBAE-G-ND@OVA treatment including the TLR and NOD-like receptor signaling pathways (Fig. 11B), with results being presented based on q-values and enrichment degree. The NOD-like receptor and cGAS-STING signaling pathways were among the most upregulated (Fig. 11C). To further confirm these results and clarify the underlying molecular mechanisms where LNT-PBAE-G-ND@OVA was able to induce macrophage activation, key cGAS-STING pathway proteins were analyzed, revealing that LNT-PBAE-G-ND@OVA upregulated cGAS, STING, TBK1, and IRF3 (Fig. 11D). These data thus confirmed the ability of LNT-PBAE-G-ND@OVA to activate cGAS-STING-TBK1-IRF3 signaling.

### 3.12 cGAS inhibitor treatment suppresses the ability of LNT-PBAE-G-ND@OVA to induce macrophage activation

**Fig. 12:**
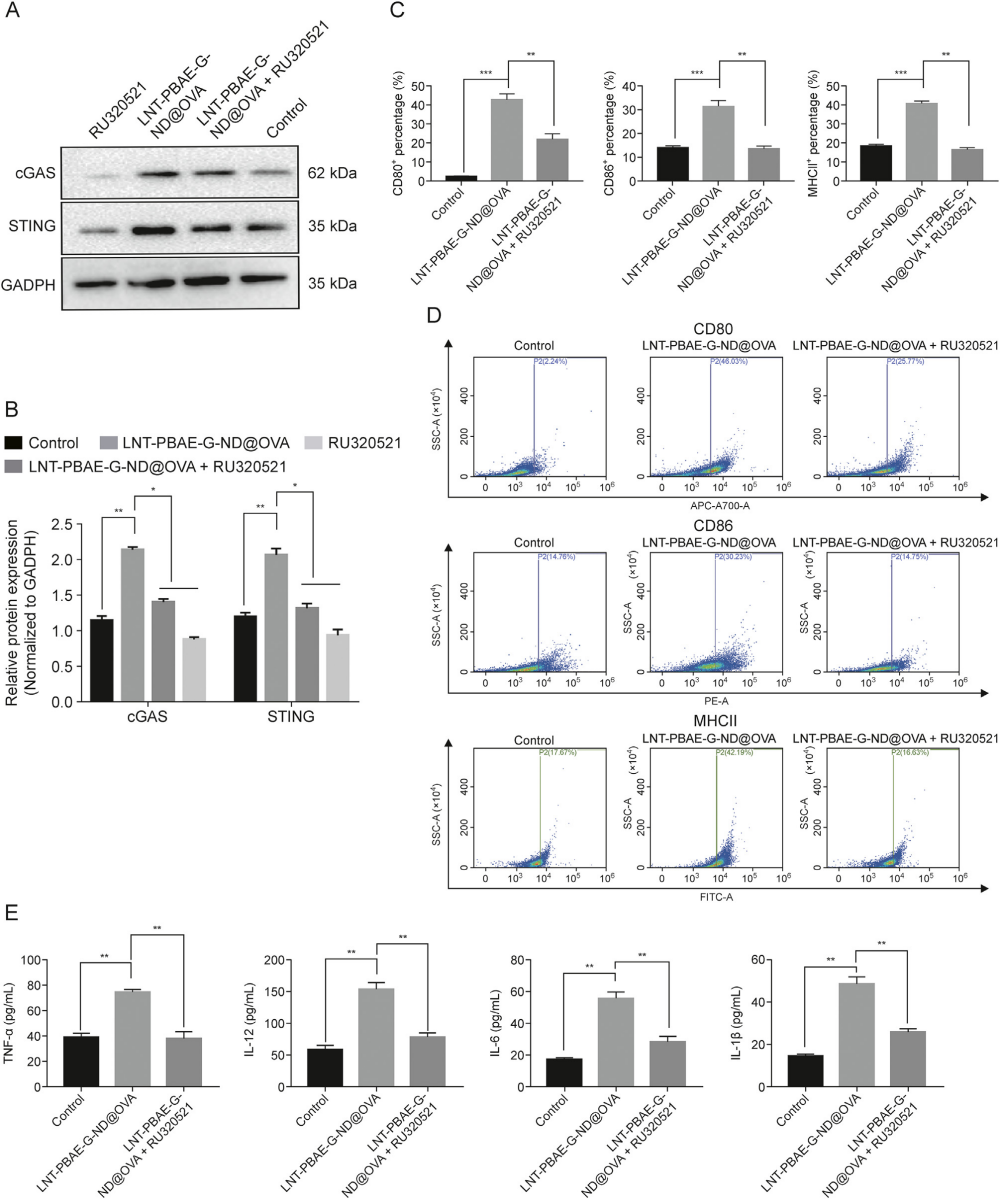
The impact of inhibiting cyclic guanosine monophosphate-adenosine monophosphate (cGAMP) synthase-stimulator of interferon genes (cGAS-STING) signaling on macrophage costimulatory molecule expression following treatment for 48 h with lentinan-functionalized poly(β-amino ester)-guanidine-phenylboronic acid-nanodiamond system was developed to carry ovalbumin (LNT-PBAE-G-ND@OVA). (A) cGAS and STING levels were measured via Western blotting. (B) Relative protein levels of cGAS-STING pathway components. (C, D) Macrophage MHCII, CD80, and CD86 expression levels (C) and representative FACS plots (D). (E) The impact of cGAS-STING pathway inhibition on macrophage secretion of interleukin (IL)-6, IL-12, IL-1β, and tumor necrosis factor-alpha (TNF-α). Data are means ± standard error of measurement (SEM), *n* = 3, ^∗^*P* < 0.05, ^∗∗^*P* < 0.01, ^∗∗∗^*P* < 0.001. GADPH: glyceraldehyde 3-phosphate dehydrogenase.

The activation of the cGAS-STING pathway is integral to innate immunity that can be activated in response to the double-stranded DNA (dsDNA) released by bacteria and viruses that can invade host cells. Such dsDNA binding results in a change in cGAS structure that catalyzes cGAMP production, with this second messenger in turn binding to STING, thereby activating it and subsequently promoting TBK1 activation. This further drives type I interferon (IFN) expression and the upregulation of a range of inflammatory cytokines capable of spurring the induction of more robust immune responses. This cGAS inhibitor RU320521 was thus next employed to example the interplay between cGAS-STING-TBK1-IRF3 signaling and the LNT-PBAE-G-ND@OVA-mediated activation and maturation of macrophages. Treatment with this inhibitor was sufficient to reduce the expression of cGAS and STING at the protein levels (Figs. 12A and B). Relative to macrophages treated with LNT-PBAE-G-ND@OVA alone, those also treated with RU320521 exhibited significantly decreased surface CD80, CD86, and MHCII expression (*P* < 0.01, Figs. 12C and D), with a concomitant drop in IL-6, IL-12, IL-1β, and TNF-α secretion by these cells (*P* < 0.01, Fig. 12E). Together, these data suggest that the cGAS-STING pathway may be required for the ability of LNT-PBAE-G-ND@OVA to readily activate macrophages.

## 4 Discussion and conclusion

Vaccination is the most common medical method and main pool to prevent chronic and infectious diseases, for which the potent and secure adjuvant platform is needed [39,40]. High effective loading rate and immune cell targeting agents were always neglected in the design of the adjuvant, which influences the contact area and multifaceted interaction between the adjuvant formulation and the APCs, and eventually impacts the initiation and activation of the immune response. Targeting strategy can be used to enhance the bioavailability and specificity of vaccine adjuvant and reduce their toxicity. Carbohydrates that have a strong affinity with sugar-binding proteins are desirable targeting groups [41]. Polysaccharides with multivalent binding capability show an even higher affinity with cellular ligands compared with their monosaccharide counterparts. Carbohydrates have also been applied to target macrophages for the delivery of immune enhancer (*e.g*, OVA) to access intracellular pathogens.

In this study, we report a dual-responsive vaccine adjuvant delivery system (LNT-PBAE-G-ND) that can encapsulate OVA immune enhancer (LNT-PBAE-G-ND@OVA), thereby effectively enhancing immune responses (Scheme 1). The NP has a core-shell structure. LNT with desirable biocompatibility forms the hydrophilic shell of the NP, and shows a strong affinity with lectins expressed by macrophage, thereby enhancing the bioavailability of the NP. A biodegradable PBAE-G-Bpolymer in the hydrophobic core can encapsulate immune enhancer, OVA. The LNT-PBAE-G-ND polymer was synthesized according to the synthesis scheme shown in Scheme 1. The hydrophilic and chimeric modified PBAEs shell of these LNT-PBAE-G-ND@OVA NPs was thus generated using a one-pot approach in this study, yielding homogenous spherical NPs as visualized via DLS and TEM with a low PDI below 0.3 (Fig. 1). There were no changes in particle size, PDI, and Zeta potential of LNT-PBAE-G-ND and LNT-PBAE-G-ND@OVA within 35 days at 37 °C, suggesting that LNT-PBAE-G-ND had great stability for 35 days. The release *in vitro* (different pHs and inflammation immune microenvironments) had illustrated that LNT-PBAE-G-ND@OVA could provide a controlled release of OVA and LNT (Fig. 2). Meanwhile, the LNT loaded on the surface of LNT-PBAE-G-ND@OVA can be more effectively taken up and presented by APC. Macrophages, as professional APCs, can effectively take up and present antigens, activating T cells to initiate adaptive immunity. LNT and OVArelease efficiency of LNT-PBAE-G-ND@OVA was evidently increased compared with PBAE-G in inflammatory environment (Fig. 3). These results described above suggested that LNT-PBAE-G-ND@OVA could serve as a great carrier to deliver the antigen and LNT, facilitating antigen transport and uptake in macrophages while enhancing the pharmacological activity of LNT and OVA (Fig. 4).

To further verify the adjuvant effect of LNT-PBAE-G-ND@OVA, the serum of the mice was collected on the appointed days to assess the production levels of antibodies, including IgG, IgG1 and IgG2a. Mice treated with LNT-PBAE-G-ND@OVA formulation produced a long-lasting and powerful antigen-specific immune response from day 28 to day 42, and the expression level of antigen-specific antibodies maintained a growth trend till day 42. The higher antibody titers in LNT/OVA and LNT-PBAE-G-ND@OVA groups may be attributed to the immune enhancement effect of LNT and OVA, which promotes phagocytosis of the LNT-PBAE-G-ND carrier.

Studies have shown that LNT and OVA regulate macrophage viability and gut microorganism, which play an immune-enhancing role. Moreover, the selective regulation of microbiota has great therapeutic potential in immune enhancement, tumor therapy, vaccination, and antibiotic resistance. To illustrate the interactions between the NP and immune enhance and investigate the underlining mechanisms of LNT-PBAE-G-ND@OVA, an RNA-seq analysis of these macrophages was performed. The results showed that NOD-like receptor signaling pathway and cGAS-STING signaling pathway were the main regulatory signaling pathways. A growing body of evidence suggests that cGAS-STING signaling pathway is closely related to the occurrence of pathogenic microbial infection, tumor and autoimmune diseases, and the abnormality of this pathway may lead to the occurrence of a variety of diseases. In this study, cGAS-STING pathway may be required for the ability of LNT-PBAE-G-ND@OVA to readily activate macrophages, which demonstrate that LNT-PBAE-G-ND@OVA may be an agonist of cGAS (Figs. 9 and 10). In addition, cGAS and STING agonists have great potential in enhancing tumor immunity and improving disease.This provides a new way to use LNT-PBAE-G-ND@OVA.

In summary, we developed a mRNA vaccine delivery system based on LNT-PBAE-G-ND. *In vitro*, LNT-PBAE-G-ND@OVA could transfect mRNA into multiple cell lines and increase the immune response. We also provided *in vivo* evidence that LNT-PBAE-G-ND@OVA/mRNA administered intramuscularly could efficiently deliver vaccine to induce potent humoral and cellular immune responses and show no obvious toxicity, which demonstrated the potential of LNT-PBAE-G-ND@OVA as a safe and effective delivery carrier for mRNA vaccine.

## CRediT authorship contribution statement

**Zhiqiang Zhang**: Conceptualization, Data curation, Methodology, Project administration, Resour-ces and Writing - review & editing. **Xia Ma:** Conceptualization, Data curation, Methodology, Project administration and Funding acquisition. **Li Wang**: Formal analysis,Investigation, and Writing - review & editing. **Hui Wang**: Funding acquisition, Validation and Visualization.

## Declaration of competing interest

The authors declare that there are no conflicts of interest.
