# Supplementary material for: Lentinan-functionalized PBAE-G-nanodiamonds as an adjuvant to induce cGAS-STING pathway-mediated macrophage activation and immune enhancement
Source: J Pharm Anal. 2023 Dec 22;14(12):100922. doi: 10.1016/j.jpha.2023.12.012 (PMC11750279; doi:10.1016/j.jpha.2023.12.012)
Supplement: Multimedia component 2 [file mmc2.docx]

Lentinan Functionalized PBAE-G-nanodiamonds as an Adjuvant to Induce cGAS-STING Pathway-mediated Macrophage Activation and immune enhancement

Authors：Zhiqiang Zhang^1^, Xia Ma^2*^, Hui Wang^1*^

1.College of Pharmacy, Henan University of Traditional Chinese Medicine, Zhengzhou, 4

450045, Henan Province, China 5

2.College of Animal Medicine, Henan University of Animal Husbandry and Economy, 6

Zhengzhou, 450046, Henan Province, China 7

Correspondence author 8

1.Hui Wang, Professor. College of Pharmacy, Henan University of Traditional Chinese 9

Medicine, Zhengzhou, 450045, Henan Province, China. Email: 10

2.Xia Ma, Professor. College of Animal Medicine, Henan Universty of Animal Husbandry 11

and Economy, Zhengzhou, 450046, Henan Province, China. Email:maxia801010@126.com 12

a These authors made equal contributions to this work.

Materials and methods

**Materials.** 2,2'-Dithiodiethanol, acryloyl chloride, N-Boc-ethylenediamine and 1H-pyrazole

1-carboxamidine were purchased from Chem-Impex Int'l. Inc. (Wood Dale, IL, USA). 4

(Hydroxymethyl)phenylboronic acid pinacol ester, triphosgene were obtained from Acros

Organics (Pittsburgh, PA, USA). Diethanolamine, diethylenetriamine and dextran was bought

from Alfa Aesar (Tewksbury, MA, USA). Other reagents were purchased from Thermo Fisher

Scientific (Fitchburg, WI, USA) and used as received unless otherwise stated.

**Characterization.** ^1^H NMR spectra of all intermediate and final polymer products were

recorded on a Bruker 400 spectrometer in D2O at 25 °C. The molecular weights (Mn and

Mw) and polydispersity indices (PDI) of the polymers were determined by a gel permeation

chromatographer (GPC) equipped with a refractive index detector, a light scattering detector,

and a viscometer detector (Viscotek, USA).FT-IR spectra were recorded on a Fourier transform infrared spectrometer (NicoletiS10, Thermo, USA) with the range of 4000-400 cm^− 1^. UV–visible spectra were recorded between 200 nm to 1000 nm wavelengths. The average diameter and homogeneous distribution of NPs were confirmed by DLS.


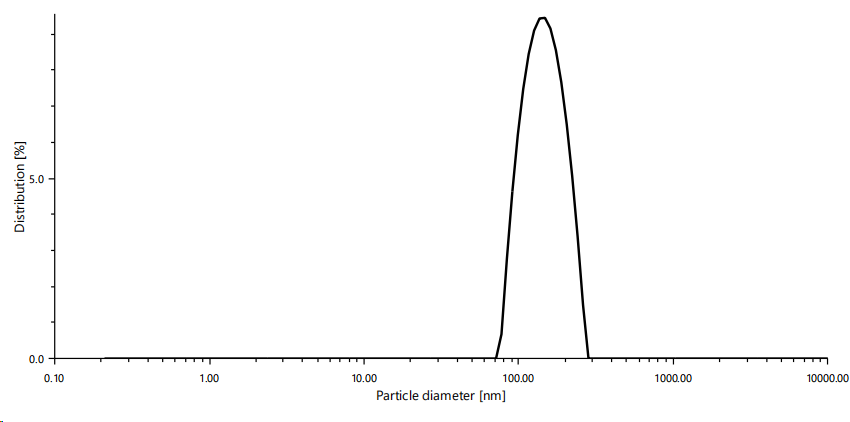
Figure S1 Average diameter of LNT-PBAE-G-ND@OVA


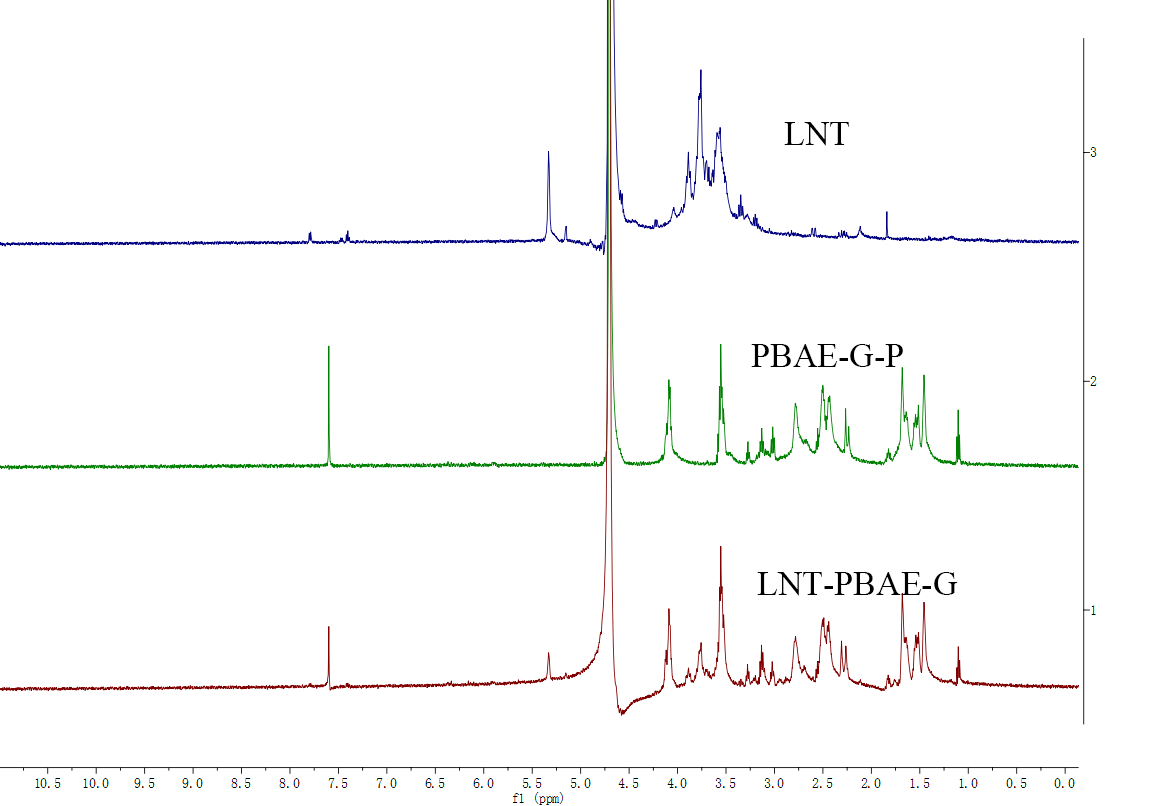


Figure S2. 1H NMR spectrum of LNT, PBAE-G-P, LNT-PBAE-G in D_2_O.


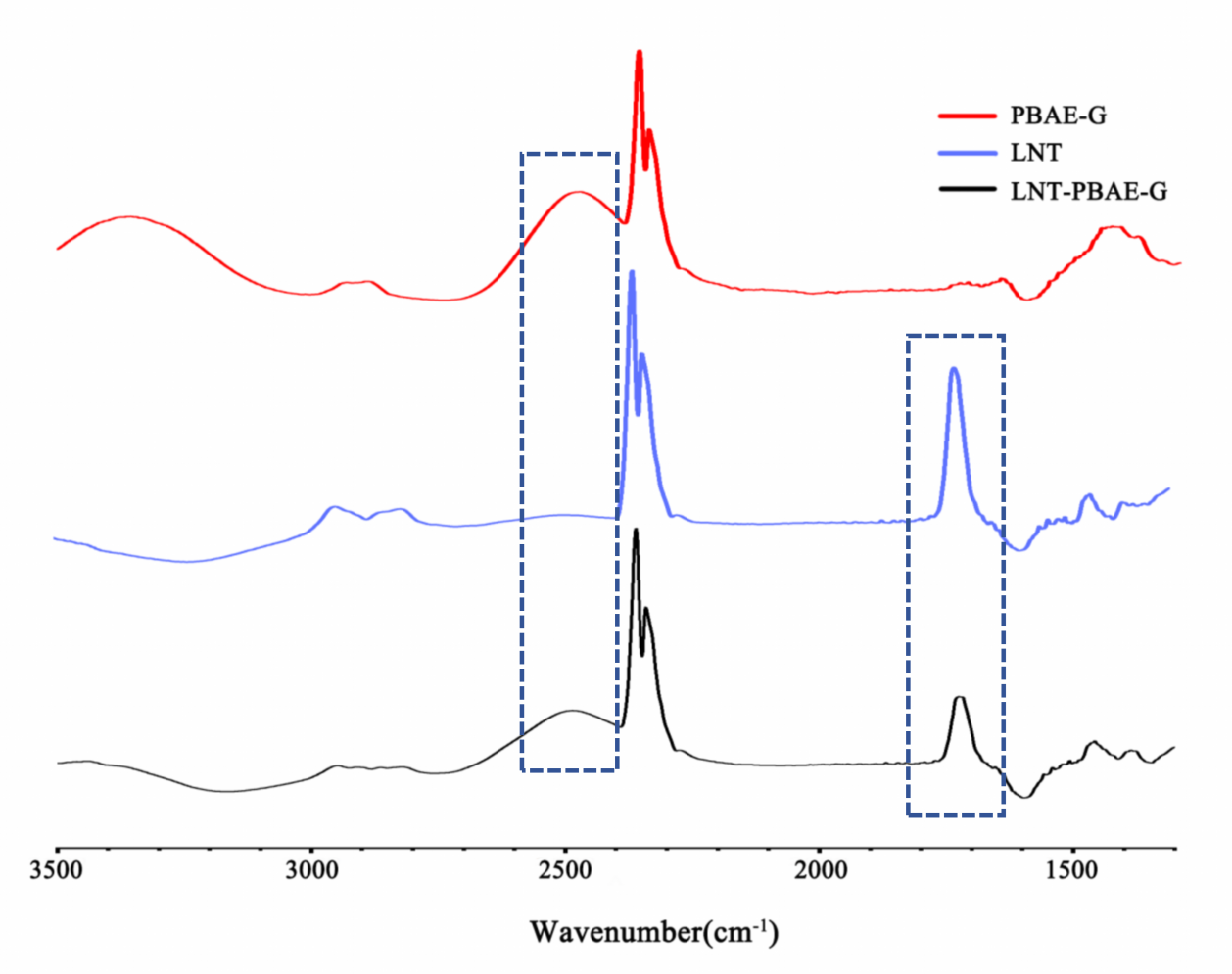


Figure S3. FT-IR of PBAE-G, LNT, LNT-PBAE-G.


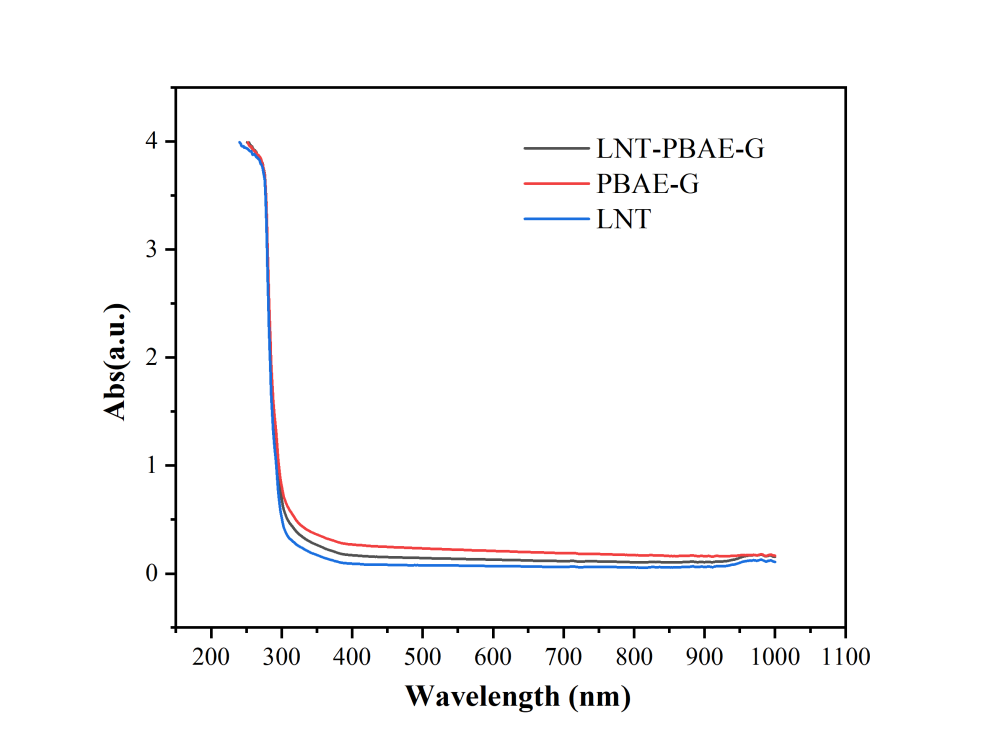


Figure S4. UV-vis of LNT, PBAE-G-P, LNT-PBAE-G .


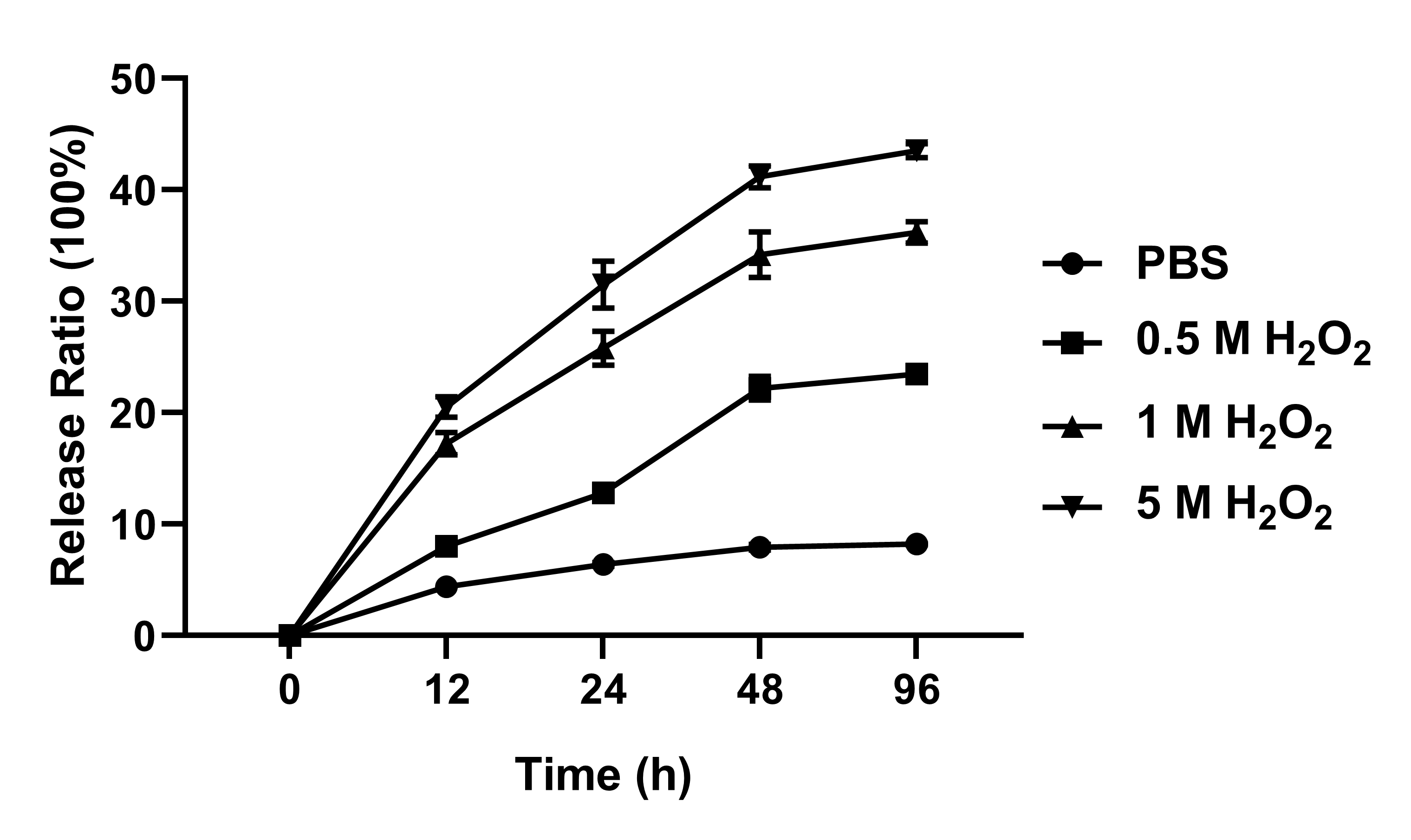


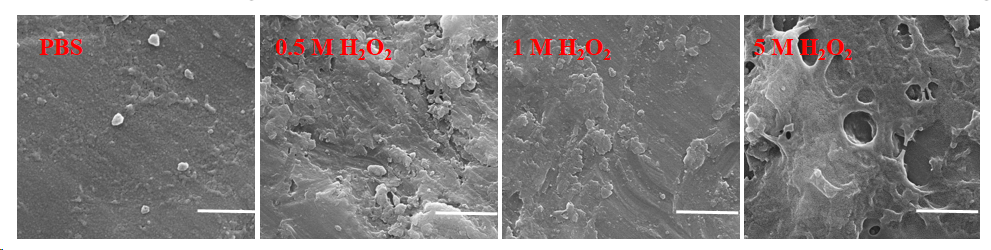


Figure S5. LNT release ratio from LNT-PBAE-G with different H_2_O_2_ .
